# A MYB Transcription Factor Atlas Provides Insights into the Evolution of Environmental Adaptations in Plants

**DOI:** 10.3390/ijms24032566

**Published:** 2023-01-29

**Authors:** Chaofan Zhang, Chen Jiao, Xuepeng Sun, Xiaolong Li

**Affiliations:** 1 Key Laboratory of Quality and Safety Control for Subtropical Fruit and Vegetable, Ministry of Agriculture and Rural Affairs, College of Horticulture Science, Zhejiang A & F University, Hangzhou 311300, China; 2 College of Agriculture and Biotechnology, Zhejiang University, Hangzhou 310058, China; 3 Collaborative Innovation Center for Efficient and Green Production of Agriculture in Mountainous Areas of Zhejiang Province, College of Horticulture Science, Zhejiang A & F University, Hangzhou 311300, China

**Keywords:** green plants, MYB gene, evolution, environmental adaptation, HGT, neo-/sub-functionalization

## Abstract

The MYB transcription factor superfamily includes key regulators of plant development and responses to environmental changes. The diversity of lifestyles and morphological characteristics exhibited by plants are potentially associated with the genomic dynamics of the MYB superfamily. With the release of the plant genomes, a comprehensive phylogenomic analysis of the MYB superfamily across Viridiplantae is allowed. The present study performed phylogenetic, phylogenomic, syntenic, horizontal gene transfer, and neo/sub-functionalization analysis of the MYB superfamily to explore the evolutionary contributions of MYB members to species diversification, trait formation, and environmental adaptation in 437 different plant species. We identified major changes in copy number variation and genomic context within subclades across lineages. Multiple MYB subclades showed highly conserved copy number patterns and synteny across flowering plants, whereas others were more dynamic and showed lineage-specific patterns. As examples of lineage-specific morphological divergence, we hypothesize that the gain of a MYB orthogroup associated with flower development and environmental responses and an orthogroup associated with auxin and wax biosynthesis in angiosperms were correlated with the emergence of flowering plants, unbiased neo-/sub-functionalization of gene duplicates contributed to environmental adaptation, and species-specific neo-/sub-functionalization contributed to phenotype divergence between species. Transposable element insertion in promoter regions may have facilitated the sub-/neo-functionalization of MYB genes and likely played a tissue-specific role contributing to sub-/neo-functionalization in plant root tissues. This study provides new insights into the evolutionary divergence of the MYB superfamily across major flowering and non-flowering lineages and emphasizes the need for lineage-/tissue-specific characterization to further understand trait variability and environmental adaptation.

## 1. Introduction

The prevailing hypothesis for the origin of plant on earth is that it began in the ocean and that diverse algae were the earliest photosynthetic life forms to appear [1]. Approximately 500 million years ago, bryophytes appeared as the first land settlers [2]. Because bryophytes have no roots, they must live in wet habitats to cope with the threat of drought. Long-term environmental selection led to continuous evolution of plants. Approximately 430 million years ago, the emergence of the vascular system successfully addressed the threat of drought on land by transporting water from plant roots to stems [3,4]. This allowed plants to expand their habitats from wet to dry land, leading to the subsequent evolution of the dwarf bryophytes into tall woody plants. In addition to the vascular system, the evolution of various leaves, reproductive systems, and flowers contributed to plant diversity on earth. The appearance of flowers, marking the establishment of the angiosperm group, was the last milestone in the development of modern plants, dividing green plants into flowering and non-flowering branches. Based on the fossil record and molecular dating, the oldest flowering plants may have appeared as early as 250 million years ago [5], followed by rapid speciation referred to as “abominable mystery” [6,7].

Genomes hold all the genetic information necessary for life; the constituent genes determine plant configurations and phenotypes. Transcription factors are DNA-binding proteins that play key roles in gene transcription; they can be thought of as “gatekeepers” that determine whether or not a gene may be expressed. The MYBs, which comprise one of the largest transcription factor families in plants, are ubiquitous among mosses, ferns, angiosperms, and gymnosperms. They are involved in the regulation of various pathways in plant growth and development, such as biotic/abiotic stresses [8,9,10], secondary vascular system development [11,12,13], and flower/fruit development [14,15,16]. Members of the MYB family encode a highly conserved MYB domain at the N-terminus [17], which typically contains ~52 amino acid residues [18]. Based on the number of adjacent MYB domain repeats, the MYB superfamily could be classified into four major subfamilies: MYB-related proteins (1R-MYB), R2R3-MYB proteins (2R-MYB), R1R2R3-MYB proteins (3R-MYB), and 4R-like MYB proteins (4R-MYB), which have one, two, three, and four MYB domains, respectively. The 2R-MYB proteins are the most abundant in plants, and their functions have been extensively investigated and verified using multi-omic and molecular approaches [19,20,21,22,23]. Sub-/neo-functionalization after gene duplication has also been observed for many MYBs [24]. For example, in *Antirrhinum majus*, the MYB genes *AmMIXTA* and *AmMYBML1*, which are derived from duplication, control the shape of petal epidermal cells and trichome formation, respectively [25,26,27,28]; overexpression of *MYB122* in Arabidopsis led to the failed rescue of *hig1-1* chemotype by its homologue *MYB51*, but caused an increase in auxin and indolic glucosinolates in the wild-type [29].

Due to technical strides in genome sequencing, hundreds of plant genomes have been sequenced, covering the major lineages of green plant kingdom. This provides an excellent opportunity to explore the origins and evolution of important plant tissues, organs, and environmental adaptations using a large and credible dataset. Recently, a comprehensive analysis of the phylogeny and functions of MYBs has been performed in brown algae and supported the independent evolution of the 2R-MYB and 3R-MYB subfamilies [30]. The 2R-MYB has also been investigated in a broader range of plants and indicates their important roles in adaptation of terrestrial environments [31,32]. Identification of a land plant-specific 2R-MYB transcription factor has provided novel insights into the origin and evolution of cuticle in early land plants [33]. Additionally, the loss or duplication of individual MYB gene has revealed the evolutionary mechanism of the flavonol regulators in the Brassicaceae [34].

Here, we generated a Viridiplantae MYB transcription factor atlas from 437 plant genome sequences. We combined phylogenomics, horizontal gene transfer, and neo/sub-functionalization analyses of members of the MYB superfamily to explore their contributions to flowering and non-flowering plant evolution, species diversification, trait formation, and environmental adaptation. Our results not only provide new insights into the evolution of MYB transcription factors but also offer a comprehensive MYB gene family database (https://github.com/cfz1998/MYB/ accessed on 3 April 2022) that will serve as a useful platform for the research community.

## 2. Results and Discussion

### 2.1. Diverse Species Selection to Explore the Evolution of Complex Morphology

**Figure 1:**
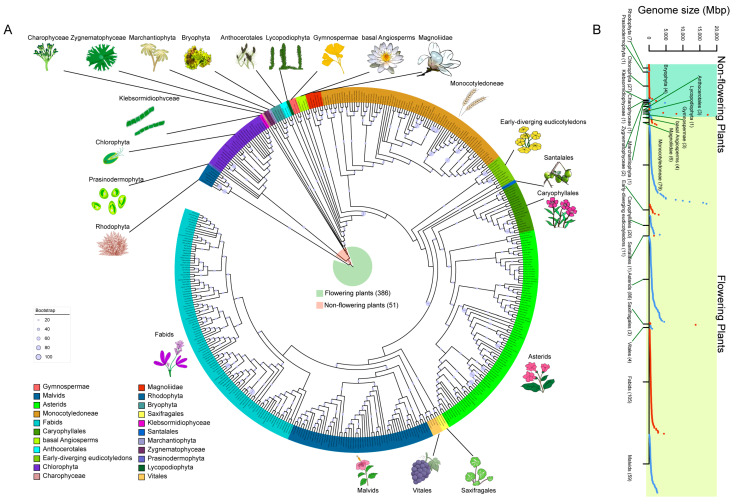
The phylogeny and genome size distribution of the 437 plant species. (**A**) Phylogenetic tree of the 437 plant species. Species were marked in different color in the outer circle to show the 22 categories (legend shown on bottom left). Representative species in each of the 22 categories was indicated and their morphology was shown. Light blue pies on the tree indicate bootstrap support of each branch with the pie size increases when bootstrap value goes high. The tree was reconstructed using the R package ‘rotl’, based on the Open Tree of Life data API. (**B**) The genome size of 437 plant species. Each point represents a species. The *y*-axis represents the genome size. Species with each of the 22 categories were ordered based on their genome size. The green background included 51 non-flowering plants and the yellow included 386 flowering plants.

A total of 693 Viridiplantae genomes and annotations were collected from various resources (Table S1). To explain the evolutionary characteristics of gene families to the greatest extent and to ensure the reliability of the analyses, we analyzed only species with complete or near-complete genome annotations. A total of 437 genomes were retained for subsequent analyses, comprising 386 flowering and 51 non-flowering plant species (Table S1; Figure 1A). To explore the evolutionary mechanisms of gene family evolution in Viridiplantae, we classified flowering and non-flowering plants into 11 categories each (for a total of 22 categories) based on plant taxonomy and the phylogenetic tree constructed in this study (Figure 1A). In non-flowering plants, species ranged from the Rhodophyta (e.g., *Cyanidioschyzon merolae*) to Gymnospermae (e.g., *Abies alba*); there were seven Rhodophyta species, one Prasinodermophyta, 27 Chlorophyta, one Klebsormidiophyceae, one Charophyceae, two Zygnematophyceae, one Marchantiophyta, four Bryophyta, three Anthocerotales, one Lycopodiophyta, and three Gymnospermae. In flowering plants, the selected species included four basal Angiosperms, six Magnoliidae, 79 Monocotyledoneae, 11 Early-diverging eudicotyledons, one Santalales, 20 Caryophyllales, 90 Asterids, three Saxifragales, four Vitales, 109 Fabids, and 59 Malvids. These species covered the major lineages of Viridiplantae, including those with and without roots in addition to terrestrial and aquatic, vascular and non-vascular, and flowering and non-flowering plants (Figure S1).

Among the species tested, most non-flowering plants had a small genome size until the emergence of gymnosperms, such as *Abies alba*, a typical representative species with a large genome size of 17.36 Gb (Figure 1B). Moreover, whole genome duplication (WGD) and polyploidization led to genome expansion in many flowering plants, such as allohexaploid wheat (17 Gb), which are proposed to facilitate environmental adaptation and speciation [35].

### 2.2. Variations in MYB Gene Number Are Associated with Plant Morphology

**Figure 2:**
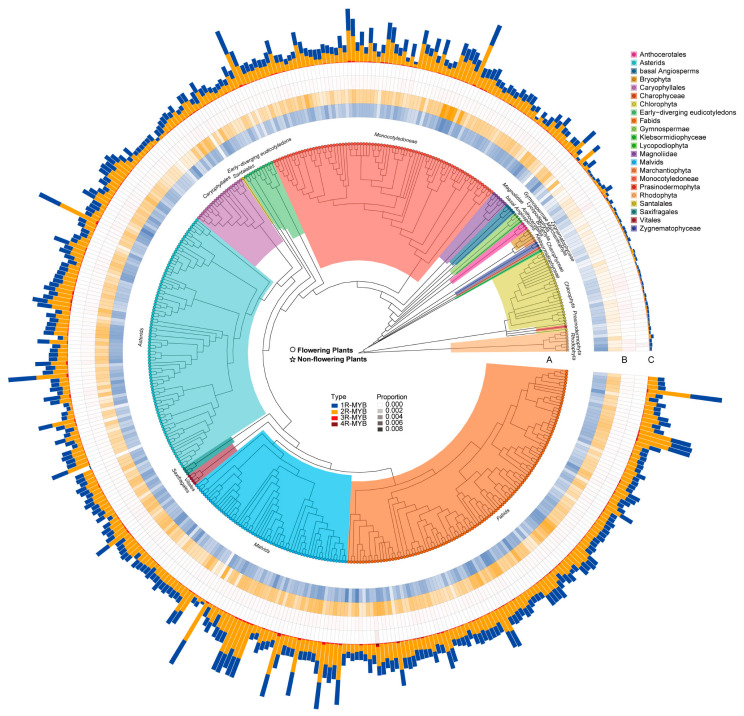
Number of MYB family members in the 437 plant genomes. (**A**) The phylogenetic tree of the 437 plant species. The tree was colored based on the information of the defined 22 categories, and the color corresponding to each category was shown on top right. Colored circle (for flowering plants) or star (for non-flowering plants) on the tip of each branch represents a species. (**B**) The four tracks next to the phylogeny show the proportion of MYB subfamily genes in the genome of each species. The tracks from inside to outside are 1R-MYB, 2R-MYB, 3R-MYB, and 4R-MYB. Each MYB subfamily (or track) was shown with a specific color. The values of percentage were divided and shown with different shade indicated in the center of the plot (dark shade means high proportion). (**C**) The outermost track shows the total number of MYB subfamily members in each genome. Blue represents 1R-MYB subfamily, orange represents 2R-MYB subfamily, red represents 3R-MYB subfamily, and dark red represents 4R-MYB subfamily. Figure was plotted using R package ‘ggtree’ (https://bioconductor.org/packages/release/bioc/html/ggtree.html accessed on 20 May 2022).

MYB transcription factors are widely involved in the regulation of plant growth and development. From the 437 selected plant genomes, a total of 113,196 MYB genes were identified. These included 2589 genes from non-flowering plants (with an average of 50.76 per genome) and 110,607 genes from flowering plants (with an average of 286.55 genes per genome). These results demonstrated that, on average, a flowering plant had nearly six times as many MYB genes as a non-flowering plant (Figure 2; Table S2). The 1R-MYB and 2R-MYB subfamilies were the main contributors to the expansion of the MYB gene superfamily in flowering plants. Thus, we speculated that MYB genes (especially 1R-MYB and 2R-MYB members) may be involved in regulating the more complex morphologies of flowering plants. The present study also observed that members of the 2R-MYB subfamily were more abundant in flowering plants, whereas members of the 1R-MYB subfamily were more abundant in non-flowering plants (Figure S2A). We additionally evaluated the degree of gene number dispersion; the coefficient of variation (*Cv*) for gene number was greater in non-flowering plants (0.98) than in flowering plants (0.49) (Table S3; Figure S2B).

We compared the number of 1R-MYB and 2R-MYB subfamily members in the 22 categories of non-flowering and flowering plants. We observed that the number of 1R/2R-MYB genes significantly increased in the Gymnospermae, a group that including perennial woody plants (Figure S3). The 1R/2R-MYB gene family was further expanded and diversified in flowering plants, which laid a solid genetic foundation for the appearance of flowering plants and species diversification. MYB gene family dispersion analysis in the 22 species categories revealed an especially high *Cv* value for the 2R-MYB subfamily in the Gymnospermae, suggesting an explosion in species diversity before the appearance of flowering plants (Figure S4; Table S4). This may have been due to a sudden change in the environment. In contrast, there were no significant differences in *Cv* observed for 1R-MYB and 2R-MYB in basal Angiosperms.

In most flowering plants, *Cv* values were higher for the 2R-MYB than the 1R-MYB subfamily, suggesting that 2R-MYB genes were more abundant in flowering plants. This was consistent with previous reports [36,37]. These results further supported the important role of 2R-MYB genes in the evolution of Angiosperms. However, we observed that the 1R-MYB family had a higher *Cv* value than the 2R-MYB family in Magnoliidae; this is generally regarded as one of the most important evolutionary nodes of extant flowering plants, with well developed perianths and apocarpous ovaries [38]. This may be due to MYB gene involvement in lignin biosynthesis, aiding in the formation of pedicels, stems, vascular bundles, or straight trunks [39]. Previous studies reported that 2R-MYB genes likely evolved from the 1R-MYB gene through duplication of an R1 repeat [40,41]. Therefore, we hypothesized that the higher *Cv* value of the 1R-MYB family in Magnoliidae indicated a potential trigger for the appearance of flowering plants, whereas the expansion of the 2R-MYB subfamily accelerated the diversification of flowering plants (Figure S4).

### 2.3. Whole Genome Duplication and Positive Selection

**Figure 3:**
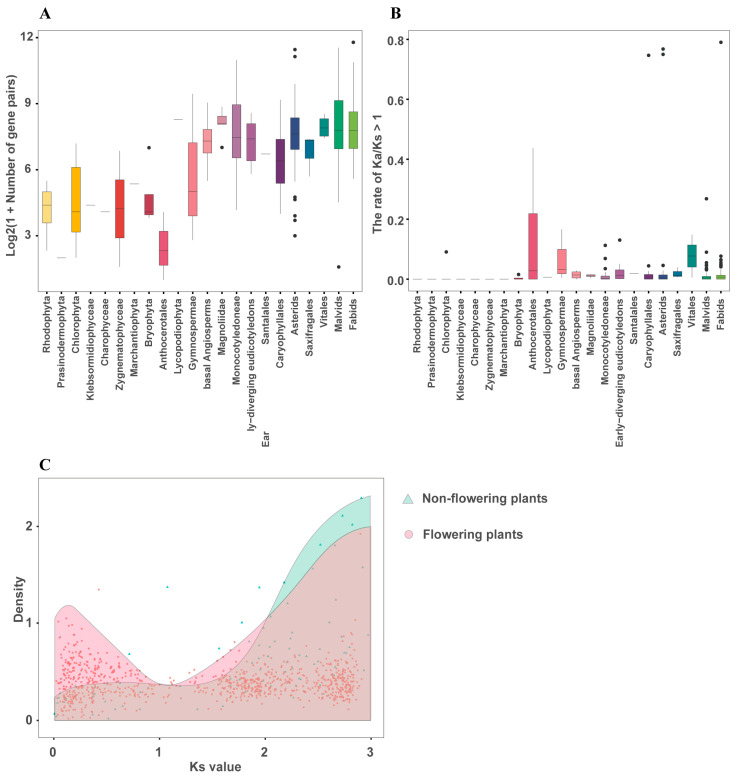
The number and selection landscape of MYB gene pairs in each of 22 defined categories. (**A**) Boxplot of the number of MYB gene pairs in the 22 defined categories. MYB gene pairs of each species were identified by orthofinder. The total number of MYB gene pairs for each plant species was used for plotting. Dark dots indicate species outliers, and the horizontal lines represent the medium of the values. (**B**) Boxplot of the proportion of MYB gene pairs in each species with nonsynonymous to synonymous substitution (Ka/Ks) value > 1. The proportion was calculated for each species and used for plotting. Dark dots indicate species outliers, and the horizontal lines represent the medium of the values. (**C**) The distribution of the mean Ks values for MYB gene pairs in flowering and non-flowering plants. The points represent the mean Ks values for MYB gene pairs of each of 437 species. The green background shows non-flowering plants, and the red background shows flowering plants. Only gene pairs with Ks value < 3 were included in these analyses.

Whole genome duplication (WGD) doubles the number of chromosomes in a genome, resulting in hundreds or thousands of gene duplicates. Duplicated genes are then either lost through genetic variation or retained through sub-/neo-functionalization, providing evolutionary potential for novel gene functions [42]. WGDs have been identified in the evolutionary history of many eukaryotes and are considered to be a major driving force in species diversification [43]. Recurrent polyploidization and rediploidization in flowering plants resulted in highly dynamic genome states, which contributed to the evolution and diversification of flowering plants [44,45]. The current study identified syntenic gene pairs within each of the 437 species investigated in this study (Table S5). The results showed that flowering plants had average MYB gene pair 10 times higher than that of non-flowering plants (Table S5). Only the Lycopodiophyta and Gymnospermae categories had relatively large numbers of MYB gene pairs; this might lay an evolutionary foundation for the appearance of flowering plants (Figure 3A). The current study find out the ratio of nonsynonymous to synonymous substitutions (Ka/Ks) to identify MYB genes that underwent positive selection, where genes with Ka/Ks values > 1 were considered to have undergone positive selection (Figure 3B; Table S6) [46].

Further, the present study observed the distribution of mean Ks values. From all 437 species, only gene pairs with Ks < 3 were retained, with the exception of all gene pairs with Ks > 3 from two green algae: *Chloropicon primus* and *Auxenochlorella protothecoides* (Table S7). The results showed that both flowering and non-flowering plants experienced ancient WGD. In flowering plants, ancient WGD has been reported in the common ancestors of the core eudicots (γ-triplication) [47], magnolias (λ event) [48] and monocots (τ event) [49], whereas WGDs in the ancient lineages of gymnosperms were also identified [50,51]. However, flowering plants have also undergone a recent doubling of MYB genes, which may have been caused by whole-genome duplications that did not occur in non-flowering plants (Figure 3C). We additionally observed a recent peak in Ks values in several non-flowering plants, such as *Porphyra umbilicalis*, *Chlamydomonas eustigma*, and *Penium margaritaceum* (Figure S5). This was consistent with previous studies reporting polyploidization in *P. umbilicalis* [52] and chromosome-level duplications in *C. eustigma* [53]. Although the *P. margaritaceum* genome has not had a recent WGD, substantial segmental gene duplications caused by highly abundant transposable elements (TEs) have been found [54]. These events could have doubled the MYB gene copy number, explaining the recent Ks peak. In summary, the Ks distribution showed the large-scale landscape of duplication events in flowering and non-flowering plants, and most were consistent with previously identified WGD events in a variety of species. These results suggested that MYB gene expansion via genome duplication and polyploidization has provided the material basis of plant evolution, and that widespread WGDs with lineage-specific patterns have contributed to genome complexity and species diversification in flowering plants.

### 2.4. Copy Number Variation of MYB Genes

**Figure 4:**
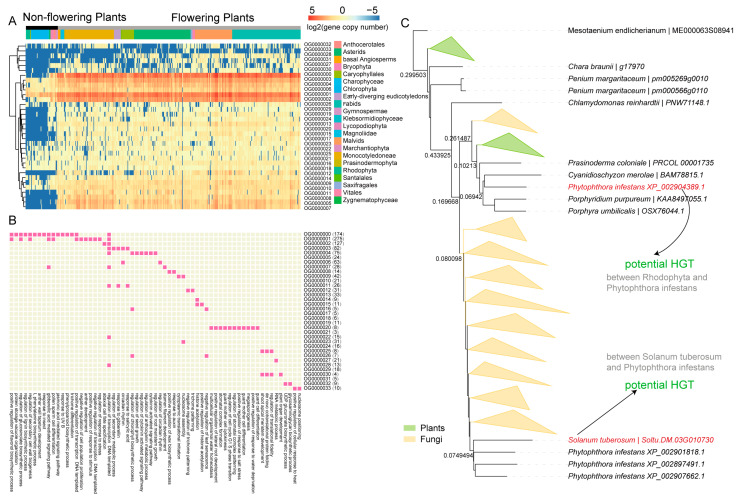
The copy number variation (CNV) and HGT of MYB genes. (**A**) A heatmap showing the gene copy number of 34 selected orthogroups in the 22 defined species categories. Each column represents a species, and each row represents an orthogroup. Colors for the 22 species categories were shown on the right. Flowering and non-flowering plants were labeled with dark and gray bars, respectively. Blue represents a low copy number (or absence) and red represents a high copy number. (**B**) Functional annotation of the selected 34 orthogroups. Each row shows an orthogroup and each column shows the functional description. Pink color marks the functional description of the corresponding orthogroup. (**C**) The phylogenetic tree of the orthogroup OG0000007. The tree was built using genes of OG0000007 and their close homologs in fungi. Colored shapes in the tree represent collapsed monophyletic lineages from plants (green) or fungi (yellow). Genes in red indicate those potentially derived from plant–fungi HGT. Number on the branch represented the local bootstrap value.

To explore the dynamic evolution of MYB genes in plants, we identified orthologous genes among 437 plant species. A total of 353 orthogroups were identified (Table S8). Most of these orthogroups were conserved between flowering and non-flowering plants, whereas 34 orthogroups had obviously different patterns (*p*-value = 2.18 × 10^−3^ on average), with higher gene copy numbers in flowering plants and fewer (or no) copies in non-flowering plants (Figure 4A; Figure S6). We therefore hypothesized that MYB genes in these 34 orthogroups were possibly important contributors to the phenotypic and adaptive evolution from non-flowering to flowering plants. We mapped each gene from the 34 orthogroups to the *Arabidopsis thaliana* protein database to identify homologous genes. A total of 1043 *Arabidopsis* genes were classified as homologs. Functional annotations indicated that these genes were involved in multiple biological processes, including responses to biotic and abiotic stresses (such as salt stress, water deprivation, cold, heat, insect feeding, and UV-B exposure), flower and seed development, root meristem growth, circadian rhythm, hormone responses, and transcriptional regulation (Figure 4B and Figure S7; Table S9).

Interestingly, OG0000008 included genes only from flowering plants, suggesting that these genes were gained after emergence of angiosperms (Figure S8A; Table S10). The genes in question were mapped to 12 homologous genes in *Arabidopsis* (Table S9); three of these (*AT1G08810*, *AT3G47600*, and *AT5G62470*) were present in most flowering plants. Previous studies have reported that *AT1G08810* (*MYB60*) was involved in response to abscisic acid, light stimulus, and water deprivation, and in wax biosynthesis, regulation of transcription, and stomatal movement [55,56,57,58]. This suggested that the three homologous genes in OG0000008 may associate with morphogenesis and environmental adaptation of flowering plants.

The present study observed that genes in OG0000000 were absent from most non-flowering plants and first appeared in Zygnematophyceae (Figure S8B; Table S10), a sister clade of the Mesotaenium, which together are recognized as the sister group to land plants (embryophytes) [59,60,61]. These genes were mapped to 170 homologous genes in Arabidopsis (Table S9), which were present in most flowering plants and were mainly involved in lignin synthesis, cell wall biogenesis, lateral root and flower development, responses to cold, salt, UV-B, and water deprivation (Table S9). These results provide evidence that the emergence of MYB genes in OG0000000 helped to increase resistance to biotic and abiotic stresses in land plants, facilitating plant terrestrialization. MYB genes in OG0000000 are comparable to previously reported crucial HGT genes obtained from soil bacteria (e.g., GRAS and PYR/PYL/RCAR) that contribute to terrestrialization [62]. A similar pattern of gene copy number in flowering plants was observed in orthogroups OG0000000 and OG0000008 (Figure S8), suggesting that an unbiased evolution of MYB genes involved in plant adaptation occurred in flowering plants.

### 2.5. The Landscape of MYB Genes Derived from Horizontal Gene Transfer (HGT)

HGT is the movement of genetic information between organisms that have mating barriers; it is a process that includes both the spread of antibiotic resistance genes among microbes and DNA sharing between microbes and plant genomes, facilitating pathogen and plant co-evolution [63]. The plant–fungi HGT events, including fungi-to-plant and plant-to-fungi transfers, have been frequently detected in *Arabidopsis thaliana*, *Oryza sativa*, *Populus trichocarpa*, *Selaginella moellendorffii*, *Sorghum bicolor*, *Peperomia polybotrya*, *Triticum aestivum*, and *Physcomitrella patens* [64,65,66]. Many resistance genes evolved long ago in natural environments with no anthropogenic influence, but these genes have rapidly spread to plant genomes [67], which may have contributed to plant adaptations to biotic and abiotic stresses in their natural environments. Fungi are among the most widely distributed microbe on Earth. They are free-living in soil or water or form parasitic or symbiotic relationships with plants [68,69] and are the causative agents of most plant diseases. To further investigate HGT events between microbe and plant genomes, we identified MYB homologs in 36 published fungal/oomycete genomes. We detected MYB genes potentially derived from plant–fungi HGT in 53 representative plants from all 22 categories (Figure S9). A total of 27,560 orthogroups were identified; 97 included homologous genes between plants and fungi/oomycete that were identified as potentially HGT-derived (Figure S10; Table S11). We manually examined the topology of the gene tree for each of the 97 orthogroups and identified high-confidence *MYB* genes from HGT events. A total of 11 high-confidence HGT events were observed, including events between *Solanum tuberosum* and *Phytophthora infestans* and between Rhodophyta and *P. infestans* (Figure 4C and Figure S11) in OG0000007. *P. infestans* belongs to the oomycete genus, a lineage of mostly filamentous eukaryotes related to diatoms and brown algae. *P. infestans* is a devastating plant pathogen that causes late blight in potato and tomato [70] and is still a major threat to food security [71]. Interestingly, except for the MYB-binding domain, the MYB genes potentially derived from HGT included a highly conserved DnaJ domain. DnaJ was originally identified as a 41-kDa heat shock protein in *Escherichia coli*; it reportedly plays important roles in protein folding and regulation of various physiological activities and also participates in several pathological processes and plant defense responses [72,73,74]. The MYB genes of potato identified as derived from HGT events will provide insights into plant–pathogen interactions, which will ultimately lead to better strategies for managing *P. infestans* blight. We also identified plant-to-fungi transfers, such as MYBS2/R-R-type MYB gene transfer from *Amaranthus hypochondriacus* and *Kalanchoe fedtschenkoi* to *Botryosphaeria dothidea*. The homologous genes of these potential HGT MYB have been reported to involve in regulation of DNA-templated transcription, response to red light, and small molecule metabolic process. Our results also suggested that MYB transcription factors are involved in plant biotic/abiotic stress responses through widespread HGT between plants and microbes; this has accelerated plant evolution and provided important gene resources and genetic bases for further resistance breeding of plants. Additional data and further experiments will be required to verify the origins of these genes in plants.

### 2.6. Sub- and Neo-Functionalization Facilitate Species Adaptation

**Figure 5:**
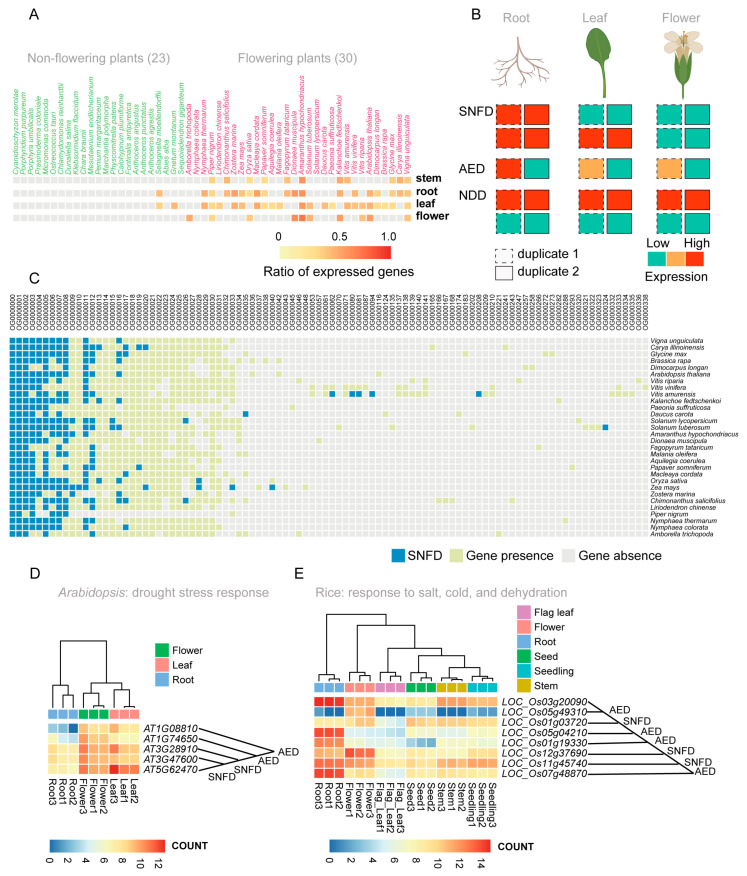
Neo-/sub-functionalization of MYB gene duplicates. (**A**) Heatmap of MYB gene expression ratios (i.e., the number of expressed MYB genes/total number of MYB genes) in root, stem, leaf, and flower of 53 selected plant species. Flowering and non-flowering plants were labeled with red and green color, respectively. Red color in heatmap indicates a high ratio, yellow indicates a low ratio, and grey indicates no published RNA-seq data. (**B**) Illustration of different expression patterns in multiple plant tissues, indicating sub-/neo-functionalized duplicates (SNFD), asymmetrically expressed duplicates (AED), and the remaining “no difference” duplicates (NDD). For the definition, SNFD indicates each gene in the duplicate pair was significantly more highly expressed than the other in at least one tissue; AEDs indicates one duplicate was significantly more highly expressed than its paralog in at least 1/3 of tissues, and its expression was not lower than its paraplog in the other tested tissues. The remaining type of duplicates were classified as NDD. (**C**) The conservation of sub-/neo-functionalized MYB genes in orthogroups of 30 selected flowering species. Blue represents SNFD, green represents gene presence but not SNFD, and grey represents gene absence. Each row represents a species, and each column represents an orthogroup. (**D**,**E**) Expression patterns of duplicate genes in Arabidopsis (**D**) and rice (**E**). Red indicates high expression and blue indicates low expression. The top row of each heatmap indicates the different tissues. The right of heatmap indicates the gene type of SNFD, AED, or NDD beside the intersection of two lines (pointing to corresponding genes).

Sub- and neo-functionalization occur after WGD events in plants and can lead to speciation or trait formation. A previous study reported that the MYB regulatory *Ruby2–Ruby1* gene cluster exhibits sub-functionalization among primitive, wild, and cultivated citrus as the regulator of anthocyanin biosynthesis [75]. Here, 53 representative plant species from 22 categories were analyzed to explore sub-/neo-functionalization of MYB genes. A total of 444 RNA-seq libraries from multiple tissues in 53 species (30 flowering plants and 23 non-flowering plants) were downloaded from publicly available databases (Table S12). Based on a threshold of reads per kilobase of transcript per million mapped reads (RPKM) > 5, we observed that more genes were expressed in the root, stem, leaf, and flower tissues (Figure 5A and Figure S12; Table S13 on GitHub) compared to other plant tissues. The gene duplicates previously identified within each of the studied plant genomes were further classified into three categories based on their co-expression patterns in different tissues of flowering plants: sub-/neo-functionalized duplicates (SNFD), in which each gene in the duplicate pair was significantly more highly expressed than the other in at least one tissue; asymmetrically expressed duplicates (AEDs), in which one duplicate was significantly more highly expressed than its sister in at least 1/3 of tissues, and its expression was not lower than its sister in the other tested tissues. The remaining duplicates were classified as having no difference (NDD) [76] (Figure 5B). The AED category had the highest ratio in nearly all 30 flowering plants (Figure S13; Table S14), suggesting the presence of a possible substitute or dosage effect in the MYB gene family. Furthermore, we explored the conservation of sub-/neo-functionalized MYB genes in orthogroups from 30 selected flowering species and observed that gene pairs in most orthogroups showed species-specific sub-/neo-functionalization (Figure 5C). Functional annotations showed that gene duplicates belonging to the SNFD and AED categories tended to be involved in biotic/abiotic stress responses, whereas gene duplicates belonging to the NDD category were mainly associated with regulation of basic plant physiological and biochemical activities (Figure S14; Table S15 on GitHub). This suggested that tissue-specific expression of MYB gene duplicates has facilitated local adaptation to different environments by plant species. We also found that gene duplicates involved in responses to environmental factors presented unbiased sub-/neo-functionalization in all of the 30 selected flowering plants (Figure 5C; Table S16).

In Arabidopsis, RNA-seq data were collected for flower, leaf, and root tissues. *AT5G62470* (*AtMYB96*) and its two paralogs, *AT3G47600* (*AtMYB94*) and *AT3G28910* (*AtMYB30*), showed the tissue-specific expression pattern that defined the SNFD category; however, it showed an expression pattern consistent with the AED category with two other paralogs, *AT1G08810* (*AtMYB60*) and *AT1G74650* (*AtMYB31*) (Figure 5D; Table S17). We observed that *AT5G62470* (*AtMYB96*), *AT3G47600* (*AtMYB94*), and *AT3G28910* (*AtMYB30*) were highly expressed in the leaf and flower, in contrast to the relatively low expression in root tissue. *AT1G08810* (*AtMYB60*) and *AT1G74650* (*AtMYB31*) were expressed at relatively low levels in the leaf and flower, but were not expressed in Arabidopsis roots. The change in tissue-specific expression patterns indicated functional diversification of *AT5G62470* (*AtMYB96*) and its paralogs. Previous studies have reported that *AT5G62470* (*AtMYB96*) plays an important role in response to drought stress mediated by abscisic acid (ABA) in Arabidopsis [23,77]. However, paralogs have developed more regulatory roles through long-term evolution (Table S17), such as responses to auxin and water deprivation, wax biosynthesis, responses to UV and absence of light, responses to cold and salt, and response to bacteria [58,78,79,80]. These results illustrate that sub-/neo-functionalization of MYB gene duplicates has contributed to the diversity of gene functions and promoted the adaptation of plants to their environments. Some reported examples of neo-/sub-functionalization of Arabidopsis MYBs were also identified in our analysis, such as *MYB12* (*AT2G47460*) and *MYB111* (*AT5G49330*), the former controls flavonol biosynthesis mainly in the root, while the latter controls flavonol biosynthesis primarily in cotyledons [81]. We also collected RNA-seq data from multiple rice tissues (flag leaf, flower, root, seed, seedling, and stem) to investigate the sub-/neo-functionalization of MYB gene duplicates. Gene *LOC_Os03g20090* was identified as potentially sub-/neo-functionalized, and is reportedly involved in salt, cold, and dehydration tolerance in rice [82]. We found that it presented unique tissue-specific expression patterns compared with its seven paralogs (Figure 5E; Table S17). Similar example cases were also identified in potato and maize (Figure S15; Table S17); in general, genes with potential sub-/neo-functionalization were mainly involved in plant development and responses to environmental stress. To further investigate the evolution of sub-/neo-functionalization, RNA-seq data for multiple tissues in non-flowering plants should be collected and analyzed in the future.

### 2.7. Transposable Elements Contributed to MYB Gene Sub- and Neo-Functionalization

**Figure 6:**
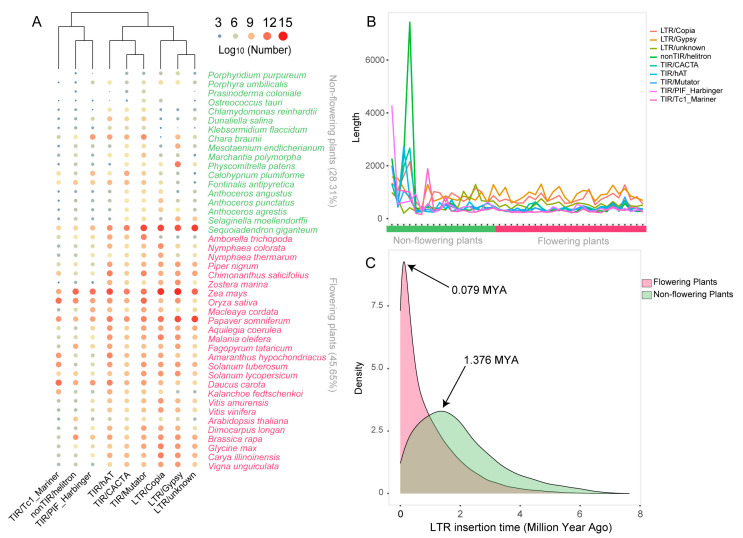
TEs have contributed to the neo-/sub-functionalization of MYB gene duplicates. (**A**) The number of different types of annotated TEs (shown on the column) in 53 representative plants from flowering and non-flowering species. Species names were shown on the right. Each row represents a species, with the green color indicating non-flowering plants and red color indicating flowering plants. Each column represents the type of annotated TEs. Color intensity and circle size correspond to the number of TEs. Larger circle and red color correspond to higher copy number of TEs. (**B**) The length distribution for different types of TEs in flowering and non-flowering plant species. Lines with different color represent different TE types. On the bottom, green bar indicates the non-flowering plants, and the red bar indicates the flowering plants. (**C**) Estimated insertion time of full-length LTR retrotransposons in flowering and non-flowering plants. Red distribution indicates flowering plants and green indicates non-flowering plants. The peak for flowering plants locates at 0.079 million years ago (MYA), and that for non-flowering plants locates at 1.376 MYA.

The complex regulatory systems of plants have enabled them to adapt to different environmental conditions continuously and rapidly. Transposable elements (TEs) are the largest component of many plant genomes, comprising ~85% of the wheat genome [83] and ~90% of the corn genome [84]. TEs therefore greatly contribute to the plasticity of plant genomes, influencing the evolution and environmental adaptation of species by generating new genetic variations and cis-acting regulatory elements through controlling the expression of nearby genes and even of unlinked inserted genes [85]. Here, to further explore the causes of sub-/neo-functionalization, we annotated TEs in the genomes of 53 representative plant species from the 22 categories. The results showed that the genomes of non-flowering plants had lower TE abundance (28.31%) and occurrence rates (3.15%) than flowering plants (45.65% and 5.07%, respectively), suggesting that an explosion of TE content occurred in gymnosperms (Figure S16; Figure S17; Table S18). Class II TEs (especially hAT, Mutator, and CACTA) were predominant in the early alga, liverwort, and bryophytes, whereas Class I elements became dominant in Selaginella, hornworts, and gymnosperms, and remained in all flowering plants (Figure 6A). In non-flowering plants, we observed a longer average TE length, although there was a large distribution (Figure 6B and Figure S18). In flowering plants, Class I TEs (LTRs), especially Gypsy, were generally longer than Class II TEs (TIRs) and fluctuated somewhat, whereas the length of Class II TEs tended to be more stable. These data suggested that Class I TEs (LTR) played a dominant role in the morphogenesis and environmental adaptation of flowering plants. An estimation of the TE insertion time indicated that the LTR expansion was relatively recent in flowering plant genomes, around 0.079 MYA, whereas a relatively more ancient LTR expansion occurred in non-flowering plant genomes, around 1.376 MYA (Figure 6C).

Further, the occurrence rate of TEs (ORT) in genic regions and the 5-Kb region up- and down-stream of MYB genes was studied in representative flowering and non-flowering plants. This analysis showed that genic regions had relatively lower ORT values compared to up- and down-stream regions. This was particularly the case in flowering plants (Figure S19), which maintained gene stability and genome plasticity. To explore the contribution of TEs to MYB gene sub-/neo-functionalization, we calculated the correlation (*R^2^*) between ORT and gene expression (RPKM > 5) in four different plant tissues. SNFD MYB genes had the highest correlation between ORT in the up-stream region and gene expression in flower (*R*^2^ = 8.20 × 10^−3^), leaf (*R*^2^ = 9.45 × 10^−3^), and stem tissues (*R*^2^ = 1.17 × 10^−2^) (Figure S20A; Table S19). These low *R^2^* values suggested that there is a non-linear relationship between them. In root tissues, TEs that occurred in genic regions more easily affected the expression and results in sub-/neo-functionalization of MYB genes (*R*^2^ = 1.67 × 10^−2^) (Figure S20B). We also observed a low correlation (*R*^2^ = 6.38 × 10^−3^) between ORT in the upstream region of AED MYB genes and expression of those genes, and a much lower correlation (*R*^2^ = 1.31 × 10^−3^) between ORT in the upstream region of NDD MYB genes and expression of those genes (Figure S20C). These results suggested that TE insertion upstream of genes may facilitate the sub-/neo-functionalization of MYB genes and that ORT plays a tissue-specific role in contributing to sub-/neo-functionalization in plant root tissue.

## 3. Materials and Methods

### 3.1. Data Collection and Preprocessing

We downloaded all available plant genomes and associated publications from the PlabiPD database (https://www.plabipd.de/plant_genomes_pa.ep accessed on 12 February 2022; version: September 2021), then retrieved the genome information for each species from the appropriate repository indicated in the corresponding literature. Genomes of 636 species and the associated annotation files were downloaded. Species without annotations were excluded from subsequent analyses. Some species did not include web links in the published articles for data retrieval. These species were searched and downloaded directly from NCBI where possible, or other databases that were publicly accessible. For species that only genome sequence and GFF annotation file were available, GFFRead software v0.12.7 [86] was used to obtain the protein and coding sequences using the command ‘gffread *. gff -g ref.fa-x cds.fa; gffread *.gff -g ref.fa -y pep.fa’. A total of 437 species with complete or near-complete genome annotations were used for subsequent analyses.

### 3.2. Phylogenetic Tree Construction

We constructed phylogenetic trees for the 437 species using two approaches (i.e., species tree from single-copy gene and existing information of phylogenetic relationship) [87]. Orthofinder [88] was used to identify single-copy genes based on protein sequences of the 437 species and to construct species trees using identified single-copy genes. Due to the large number of species, this method involved a prohibitively long computation time. We therefore used the R package “ROTL” [89], which is based on the “Open Tree of Life” API [90], to obtain a preliminary phylogenetic tree based on species names. We manually checked the resulting phylogenetic tree and used custom python scripts to remove redundant nodes and normalize node names. Consequently, we compared the tree with the Angiosperm Phylogeny Group (APG) IV system to obtain the final phylogenetic tree for the 437 studied species. Based on the phylogenetic tree, we classified the species into two main groups, flowering, and non-flowering plants. These were further subdivided into 22 categories; for non-flowering plants, which included Rhodophyta, Prasinodermophyta, Chlorophyta, Klebsormidiophyceae, Charophyceae, Zygnematophyceae, Marchantiophyta, Bryophyta, Anthocerotales, Lycopodiophyta, and Gymnospermae; whereas for flowering plants, the groups were basal Angiosperms, Magnoliidae, Monocotyledoneae, Early-diverging eudicotyledons, Santalales, Caryophyllales, Asterids, Saxifragales, Vitales, Fabids, and Malvids.

### 3.3. MYB Gene Family Identification and Classification

We identified members of the MYB transcription factor families in the 437 species from the protein sequence files. Prior to identification, we renamed and removed the isoforms from the downloaded protein sequence. For species in which proteins could be clearly distinguished based on the ID numbers, we used custom Python scripts to extract the longest transcript for each protein for subsequent analyses (https://github.com/cfz1998/MYB accessed on 3 April 2022). However, for species with customized protein sequence IDs, we could not directly establish the corresponding gene for each transcript. In those cases, we combined the GFF annotation files and retained the longest transcript.

Moreover, we downloaded the MYB conservative structure domain hidden Markov model (HMM: PF00249) seed file from the Pfam database (http://pfam.sanger.ac.uk/ accessed on 13 March 2022) and used hmmsearch software v3.3.2 [91] with an E-value of 1 × 10^−5^ to search the preprocessed protein sequence files of each species. Protein sequences that met the filter threshold were designated as candidate MYB transcription factor family members. Subsequently, we used hmmscan for further verification of the protein sequences extracted in the previous step with the E-value set to 1 × 10^−5^; sequences containing the “Myb_DNA-binding” domain were considered credible members of the MYB transcription factor family (https://data.mendeley.com/drafts/5bkpr8n2gd accessed on 18 June 2022). Further, we classified the identified MYB family members into four subfamilies based on the number of MYB domains contained in each: 1R-MYB, 2R-MYB, 3R-MYB, and 4R-MYB, where the number before “R” represents the number of MYB domains in the subfamily.

### 3.4. Duplication Detection and Positive Selection

Using the protein sequences of the MYB transcription factor family identified in 437 species, Orthofinder software v2.5.4 was used to identify orthogroups among species. For each orthogroup, we extracted the intra-species gene pairs of each species and used KaKs_Calculator 2.0 [92] to calculate Ka (non-synonymous substitution rate), Ks (synonymous substitution rate), and Ka/Ks values. Ka/Ks > 1 indicates positive selection, Ka/Ks < 1 indicates negative selection, and Ka/Ks = 1 indicates neutral selection.

### 3.5. Gene Copy Number Variation Based on Orthogroups

Orthofinder was used to identify orthologous gene clusters of MYB proteins in the 437 species and to calculate gene copy number variation with default parameters. We used Wilcox-test to evaluate the significant difference of gene number between flowering plants and non-flowering plants in each of 353 orthogroups. To explore the gene function in each orthogroup, we mapped each gene to the annotated genes in Arabidopsis using BLASTP with an E-value threshold of 1e-05 and obtained functional annotation information from the Arabidopsis Information Resource (TAIR) database (https://www.arabidopsis.org/tools/bulk/go/index.jsp accessed on 20 July 2022) [93]. Gene ontology (GO) enrichment analysis was performed using Web Crawler (https://www.webfx.com/blog/internet/what-is-a-web-crawler/ accessed on 20 July 2022) and the python module “selenium” (https://www.selenium.dev/ accessed on 20 July 2022) to extract the GO Term Enrichment results from the TAIR database (https://www.arabidopsis.org/ accessed on 20 July 2022).

### 3.6. Detection of Potential HGT

We downloaded 36 fungal genomes and the associated protein sequences from the NCBI database. Combined with 53 published plant genomes from our 22 categories, First, Orthofinder was used to obtain the single copy genes in these 89 species and to construct phylogenetic trees. Orthofinder was also used to detect the genes for which potential HGT occurred between plants and fungi/oomycete. The gene tree was verified by constructing maximum likelihood phylogeny with different models (JTT, LG, and WAG) [62]. In addition, all candidate MYB genes with potential HGT occurrences were further validated via BLASTP [94] against the NCBI non-redundant protein (NR) database. If the top matches for a candidate HGT gene in the NR database were primarily from fungi, that candidate gene was considered to have been acquired via HGT from a fungal genome.

### 3.7. RNA-seq Data Collection and Sub-/Neo-Functionalization Analysis

For the 53 selected species described above, we downloaded transcriptome datasets (for which data from at least two different plant tissues were available) from the Sequence Read Archive (SRA, https://www.ncbi.nlm.nih.gov/sra accessed on 20 May 2022). Transcriptome data from multiple plant tissues but a single project each were selected and downloaded. After obtaining the SRA number of the sequencing data, we directly downloaded the data using the “prefetch” command in SRAtoolkit (https://hpc.nih.gov/apps/sratoolkit.html accessed on 20 May 2022). Most flowering plants had transcriptome data available for different tissues, which were used to perform subsequent neo-/sub-functionalization analyses. However, RNA-seq data for non-flowering plants were very limited. From the downloaded transcriptome data, we calculated the RPKM values for each gene to indicate the relative expression level. First, fastq-dump was used to convert the original SRA file into fataq format; fastp (https://github.com/OpenGene/fastp accessed on 21 May 2022) was then used for quality control to obtain cleaned data with default parameters ‘fastp -i sample.in.R1.fq.gz -I sample.in.R2.fq.gz -o sample.out.R1.fq.gz -O sample.out.R2.fq.gz’. We then indexed the reference genome of each species using hisat2-build (hisat2-build -p 40 genome hisat2.index) and mapped the previously obtained reads data to the reference genome using Hisat2 software v2.2.1 with the default parameters: ‘hisat2 -x hisat2.index -p 4 -1 sample.out.R1.fq.gz-2 sample.out.R2.fq.gz-S sample.sam’ (http://daehwankimlab.github.io/hisat2/ accessed on 21 May 2022) [95]. For alignment results, featureCounts [96] was used to count the number of reads mapping to each gene and to obtain a file containing the number of reads for each gene using the command ‘featureCount -T 4 -p -t CDS -g gene_id -a gtf -o sample.txt sample.bam’. A custom Python script was used to calculate RPKM values based on the mapped read count. For species with RNA-seq data for multiple tissues, we used the R package ‘EdgeR’ [97] to identify differentially expressed genes (DEGs) between tissues. The thresholds of FDR < 0.01 and fold change > 2 were applied to define DEGs.

Orthofinder was used to identify high-confidence paralogous genes within each species. DEG analyses among the duplicate gene pairs for each tissue were performed using DESeq2 with a false discovery rate (FDR) cut-off of 0.05 and a fold change (FC) cut-off of 2. We then classified duplicates into three categories based on their co-expression patterns in different tissues: Sub-/neo-functionalized duplicates (SNFD), in which each gene in the duplicate pair was significantly more highly expressed than the other in at least one tissue; asymmetrically expressed duplicates (AEDs), in which one duplicate was significantly more highly expressed than its sister in at least 1/3 of tissues, and its expression was not lower than its sister in the other tested tissues. The remaining duplicates were classified as having no difference (NDD) [76].

### 3.8. Transposable Element Annotation and Insertion Time Estimation

To explore critical events at different evolutionary nodes, we selected 53 representative species from the 22 plant categories for TE analyses. By integrating homology searches and ab initio predictions, EDTA pipeline (https://github.com/oushujun/EDTA accessed on 6 June 2022) was used to identify TEs in the 53 species using the following parameters: ‘Edta.pl --genome genome.fa --species other --step all -t 20 --overwrite 0 --anno 1 --evaluate 1′. We then classified TEs as long terminal repeat retrotransposons (LTRs), terminal inverted repeats (TIRs) transposons, or Helitrons. The occurrence rate of TEs was calculated as the total length of the TE (or overlapping length) divided by the total genome sequence (or gene) length. In addition, we extracted intact LTR transposons from the “*EDTA.intact.gff3” GFF files for each species and calculated the insertion time for each LTR using the following formula:

T = K/2µ = (1 − identity)/2µ

where µ is the mutation rate of each species in units of bp per year. A general µ value of 1 × 10^−8^ was used. Results were visualized using the R package ‘ggplot2′.

## 4. Conclusions

Evolution of plants towards terrestrialization and environmental adaptation is accompanied with the expansion and neofunctionalization of important gene families particularly transcription factors [54]. In this study, we identified major changes in the presence and absence variation and copy number variation within and between subclades across flowering and non-flowering plants and discussed how these changes may have contributed to local adaptation in different environments and the related evolution of lineage-specific traits. Additionally, sub-/neo-functionalization of MYB gene duplicates has contributed to environmental adaptation, and that transposable element insertion upstream of MYB genes likely facilitated sub-/neo-functionalization. Several potential functional genes with sub-/neo-functionalization were found in Arabidopsis, rice, potato, and maize. In conclusion, this study explored the evolutionary divergence of the MYB superfamily across major flowering and non-flowering lineages of green plants and their roles in environmental adaptation through a systematic phylogenomic approach. Candidate genes identified in this study can be researched for crop improvement against abiotic stresses in the future.

## Data Availability

The original contributions presented in the study are included in the article/supplementary material, and further inquiries can be directed to the corresponding authors.

## Supplementary Materials

The following supporting information can be downloaded at: https://www.mdpi.com/article/10.3390/ijms24032566/s1.
