# Supplementary material for: A MYB Transcription Factor Atlas Provides Insights into the Evolution of Environmental Adaptations in Plants
Source: Int J Mol Sci. 2023 Jan 29;24(3):2566. doi: 10.3390/ijms24032566 (PMC9916579; doi:10.3390/ijms24032566)
Supplement: Supplementary file 1 [file ijms-24-02566-s001.zip › Supplementary Figures S1-S20.pdf]

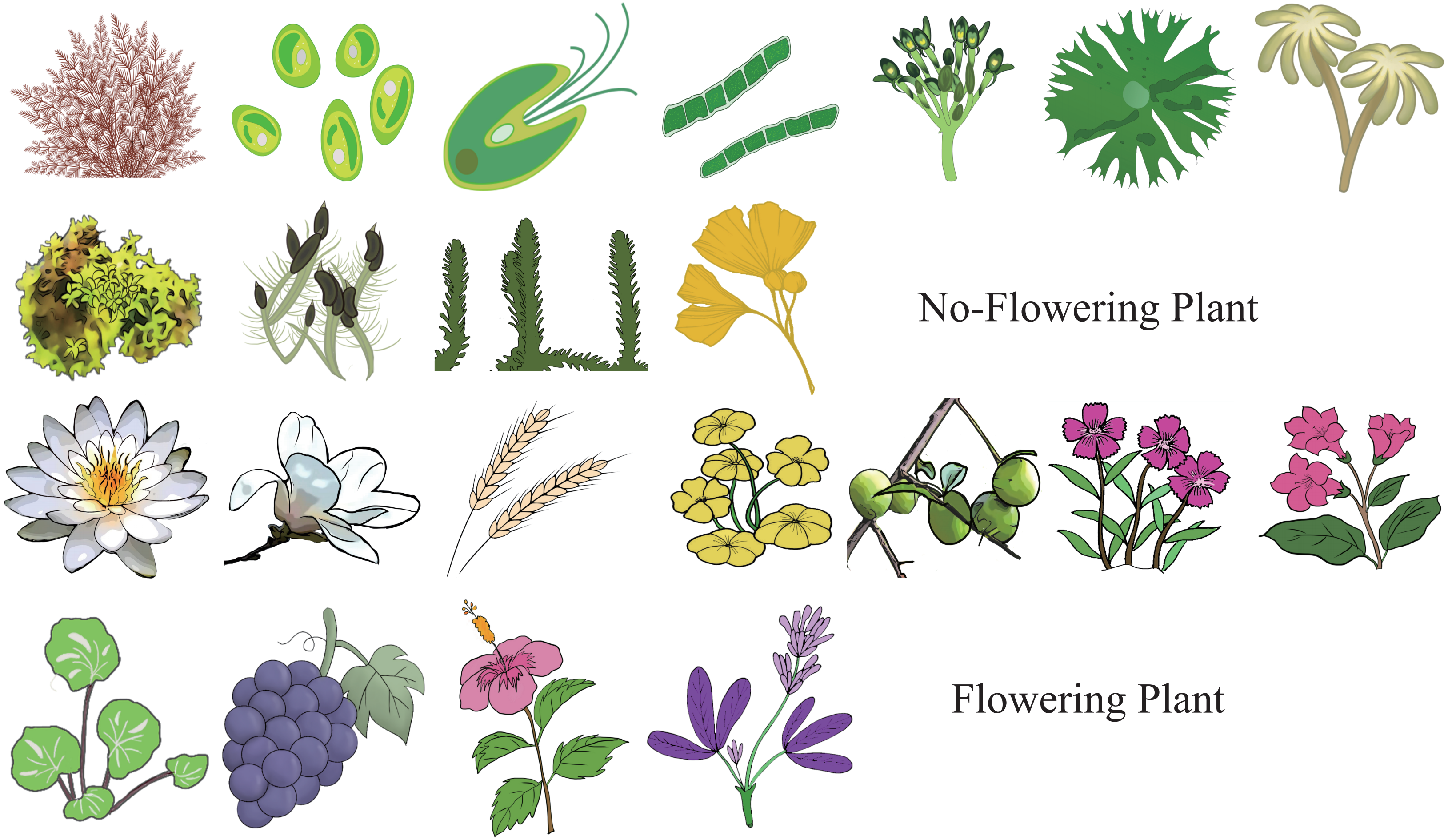

Figure S1 Morphology of representative species of 22 different categories, including 11 non-flowering plants and 11 flowering plants. In non-flowering plants, Rhodophyta, Prasinodermophyta, Chlorophyta, Klebsormidiophyceae, Charophyceae, Zygnema-tophyceae, Marchantiophyta, Bryophyta, Anthocerotales, Lycopodiophyta and Gymno-spermae were included. In flowering plants, basal Angiosperms, Magnoliidae, Mono-cotyledoneae, early-diverging eudicotyledons, Santalales, Caryophyllales, Asterids, Sax-ifragales, Vitales, Fabids and Malvids were included.

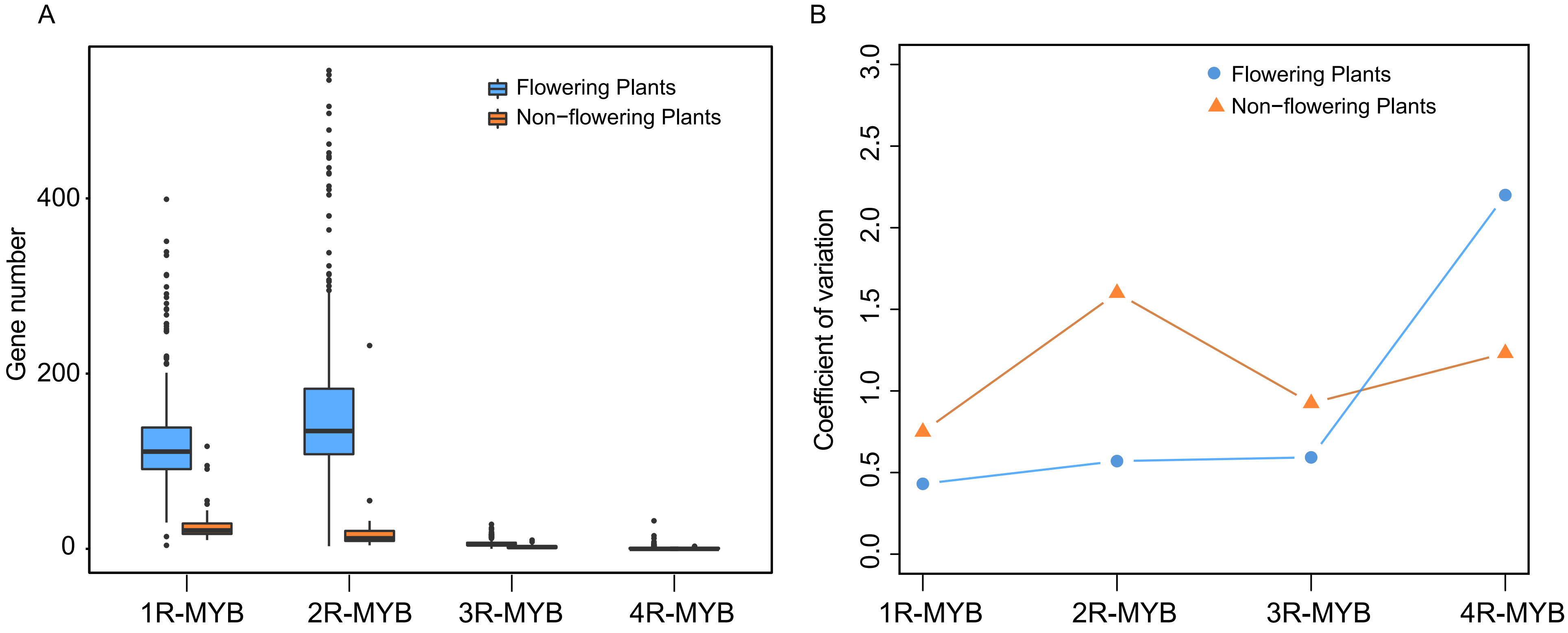

Figure S2 Identification of four MYB subfamilies, 1R-MYB, 2R-MYB, 3R-MYB, and 4R-MYB, in flowering and non-flowering plants. (A) The boxplot displays the number of members from four MYB subfamilies in flowering and non-flowering plants. Blue box represents flowering plants and orange represents non-flowering plants. (B) The line chart displays the coefficient of variation of gene number from four MYB subfamilies in flowering and non-flowering plants. Blue line with dot represents flowering plants and orange line with triangle represents non-flowering plants. R package “ggplot2” was used to plot them.

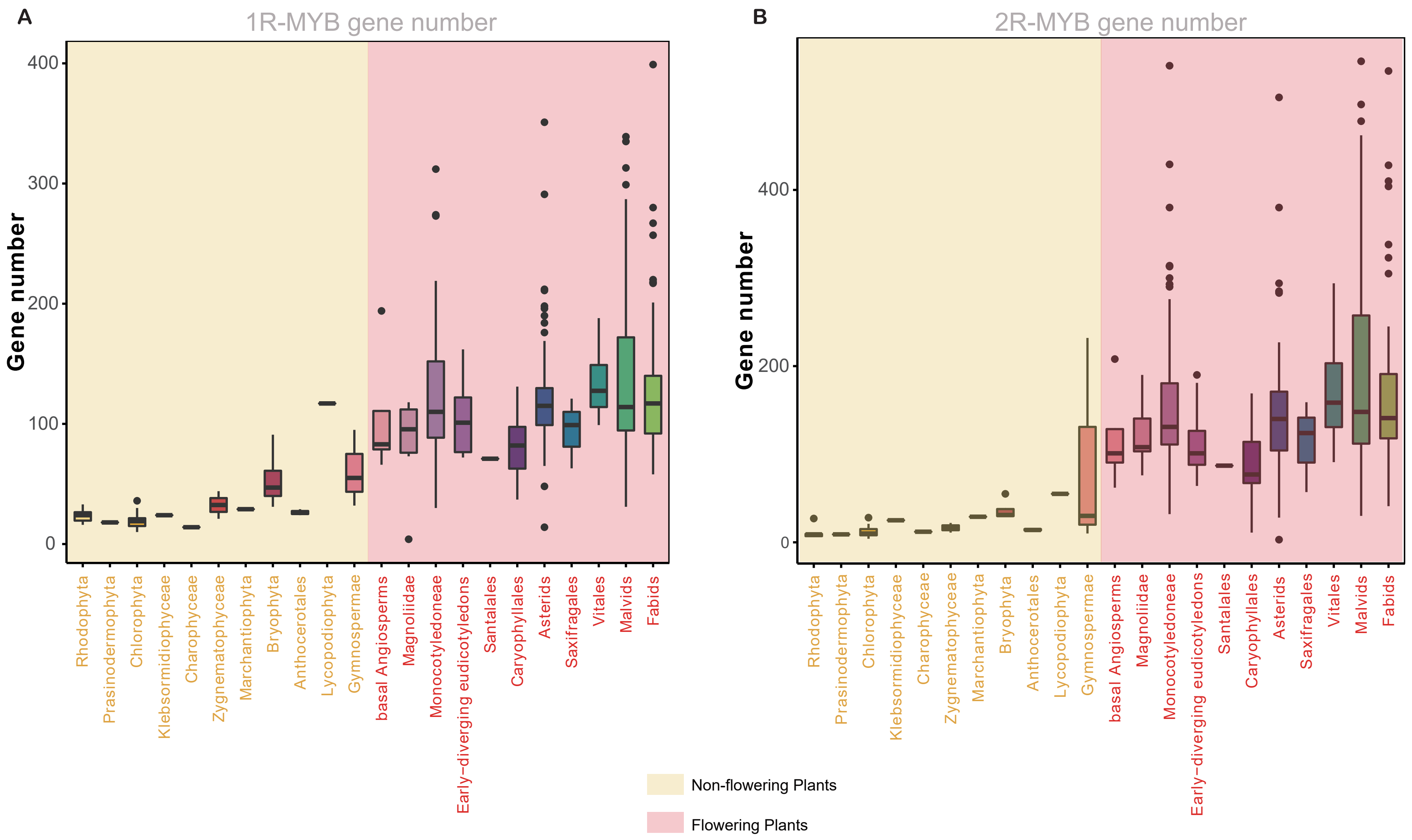

Figure S3 The number of 1R-MYB and 2R-MYB subfamilies. Each point represents a plant species. X-axis represents different categories, and y-axis represents gene number. (A) The boxplot displays the number of members from 1R-MYB subfamilies in 22 cate-gories species; (B) The boxplot displays the number of members from 2R-MYB subfami-lies in 22 categories species. Yellow background indicates non-flowering plants, and pink background indicates flowering plants.

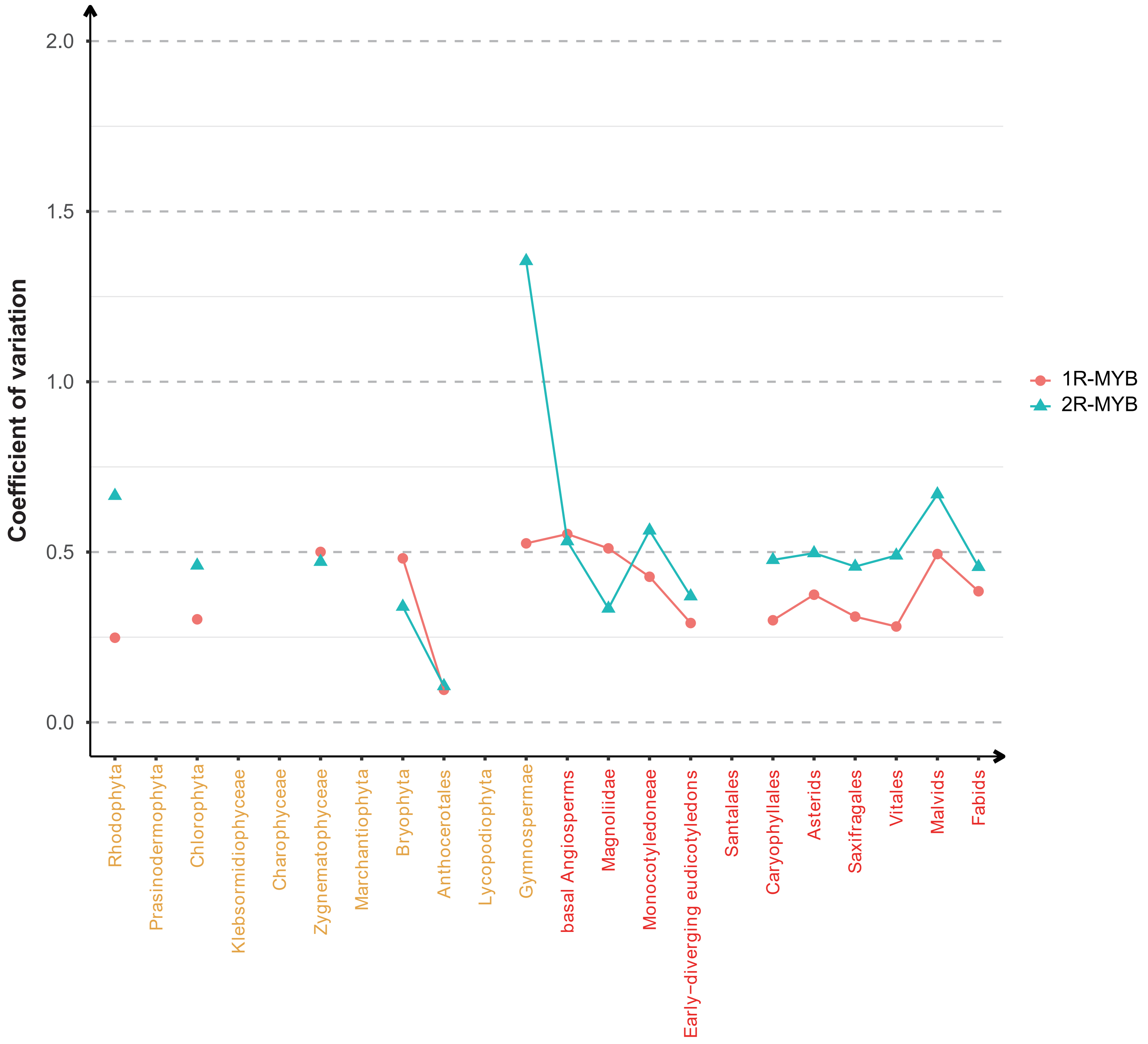

Figure S4 The divergence of gene count of 1R-MYB and 2R-MYB subfamilies in each of the 22 defined categories. The line chart displays the coefficient of variation of gene count for 1R-MYB subfamilies (red line) and 2R-MYB subfamilies (blue line). R package “ggplot2” was used to plot them.

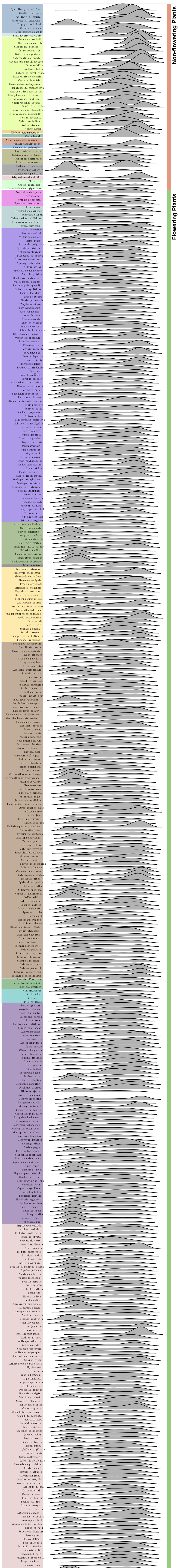

Figure S5 Distribution of synonymous substitution (Ks) value of MYB gene pairs in the 437 species. The x-axis represents the mean Ks value, and the y-axis represents the density of gene pairs from each of the 437 species.Colored boxes in the left indicate the different categories of the 437 species.

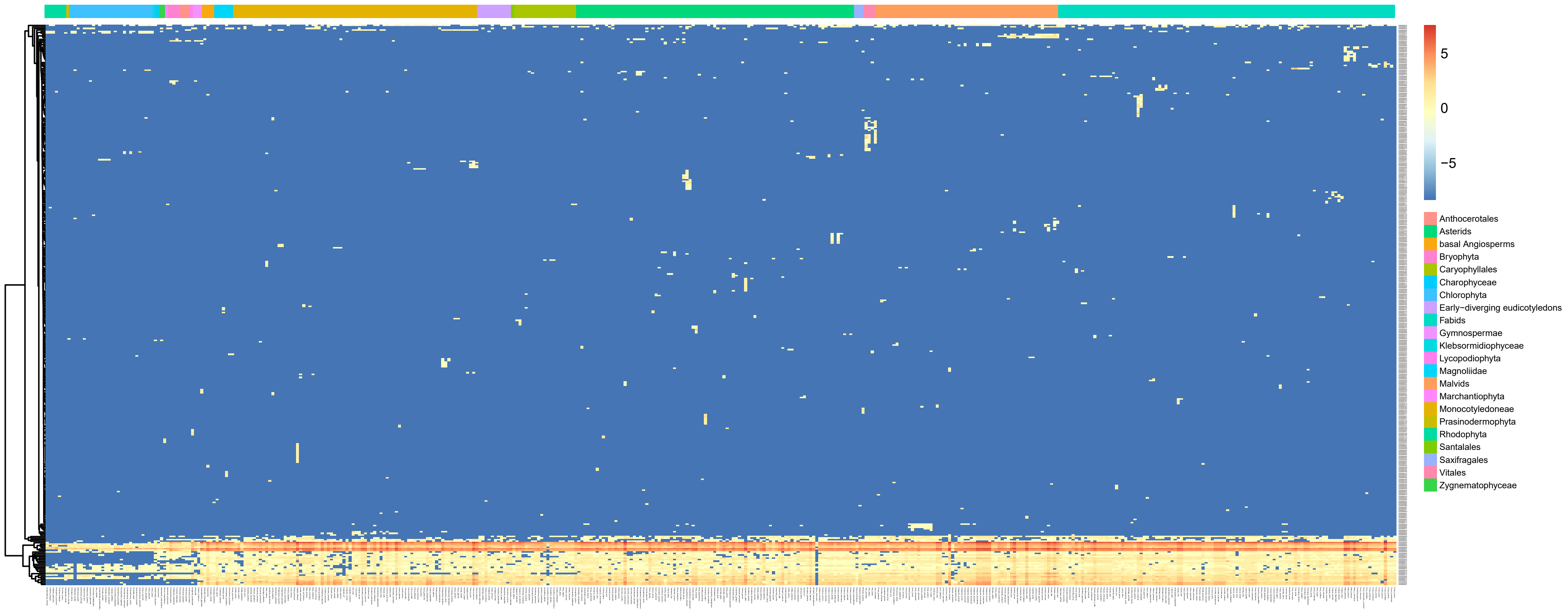

Figure S6 Heatmap of gene count of each species in the 353 orthogroups. Gene count was log2 transformed, and the red color indicates high number of gene count while the blue represents low gene count. Each column represents a plant species and each row represents an orthogroup. The different color bars in the top indicate the different species categories. “pheatmap” package in R was used to plot it.

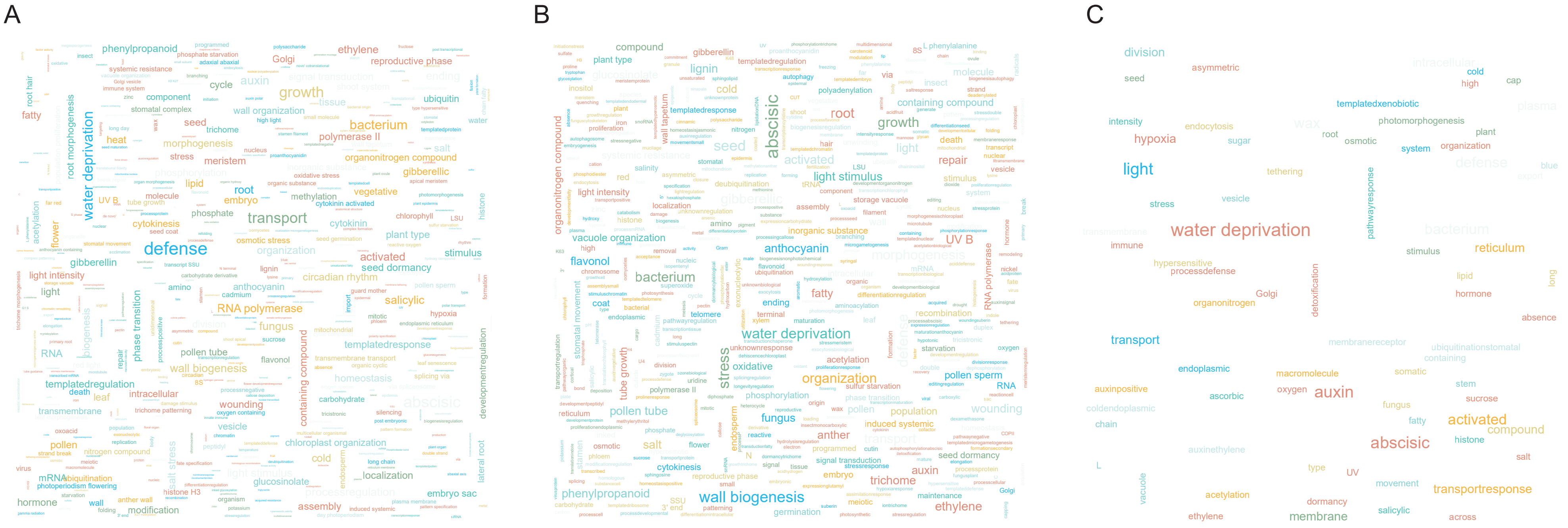

Figure S7 The word cloud figure showing functional annotation of Arabidopsis homolog from all orthogroups (A), OG0000000 (B), and OG0000008 (C). The font size correlates with the frequency of occurrence.

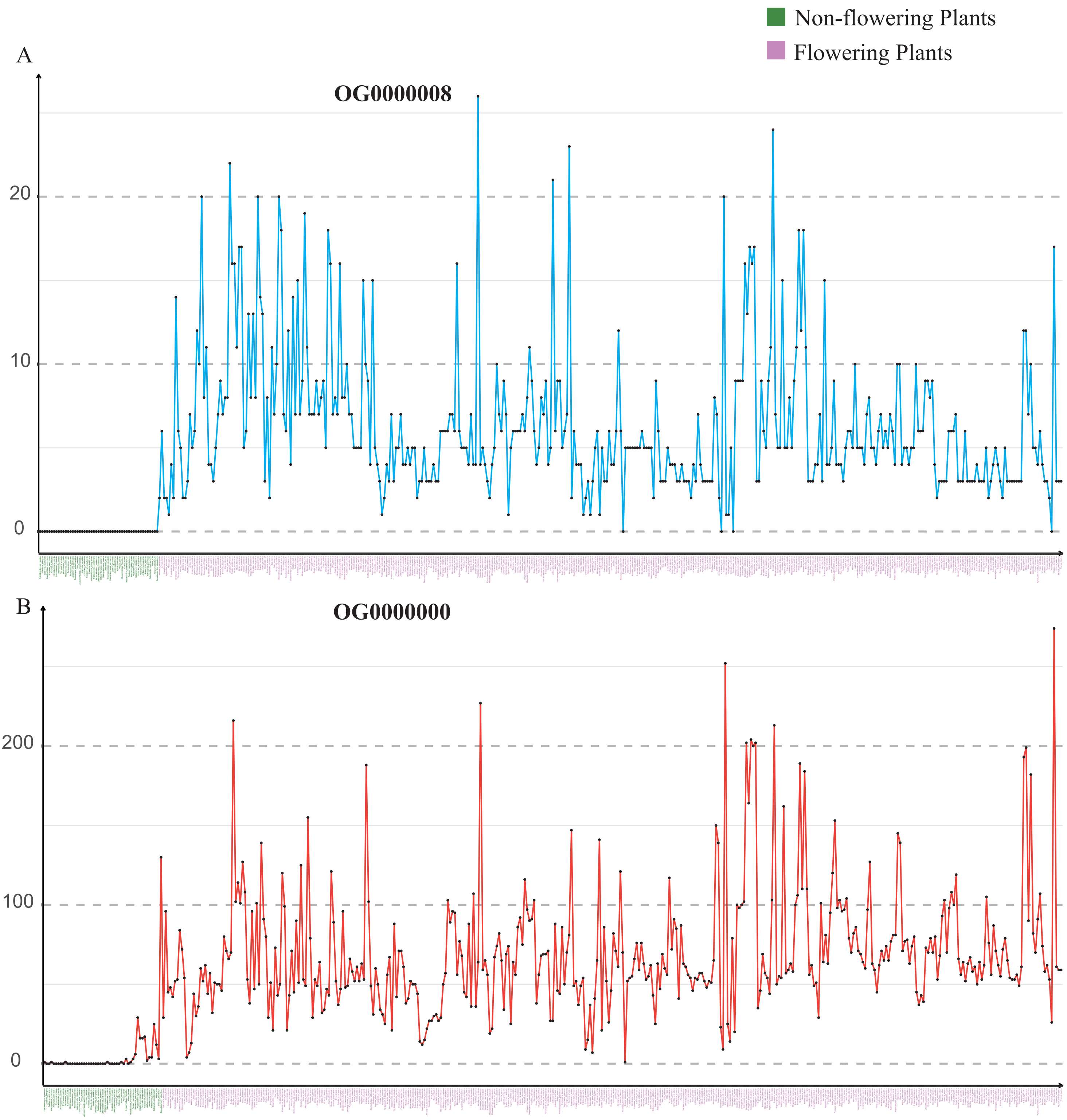

Figure S8 Gene count of orthogroup OG0000000 and OG0000008 in each species from flowering and non-flowering plants. The Y-axis represents the number of genes, and x-axis represents the different species (green text represents non-flowering plants, and pink text represents flowering plants). The upper panel with blue line represents gene count in OG0000008, and lower panel with red line represents gene count in OG0000000.

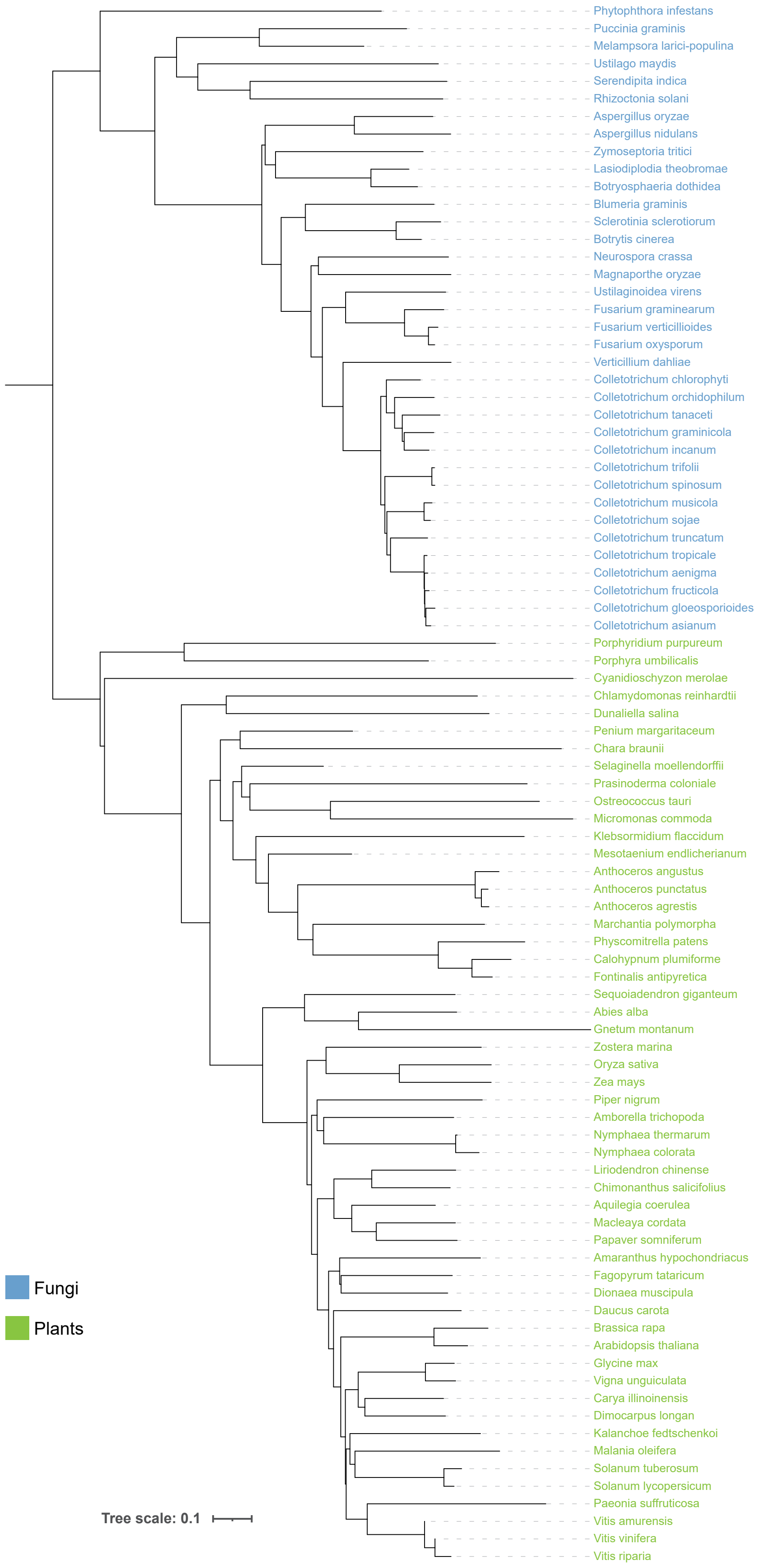

Figure S9 The phylogenetic tree of 36 fungi and 53 plants. The protein sequences of the 89 genomes were used cluster orthogourps, and the tree was reconstructed using single-copy MYB genes of all species. The species name with blue color beside the tree represents fungi and the green represents plant species.

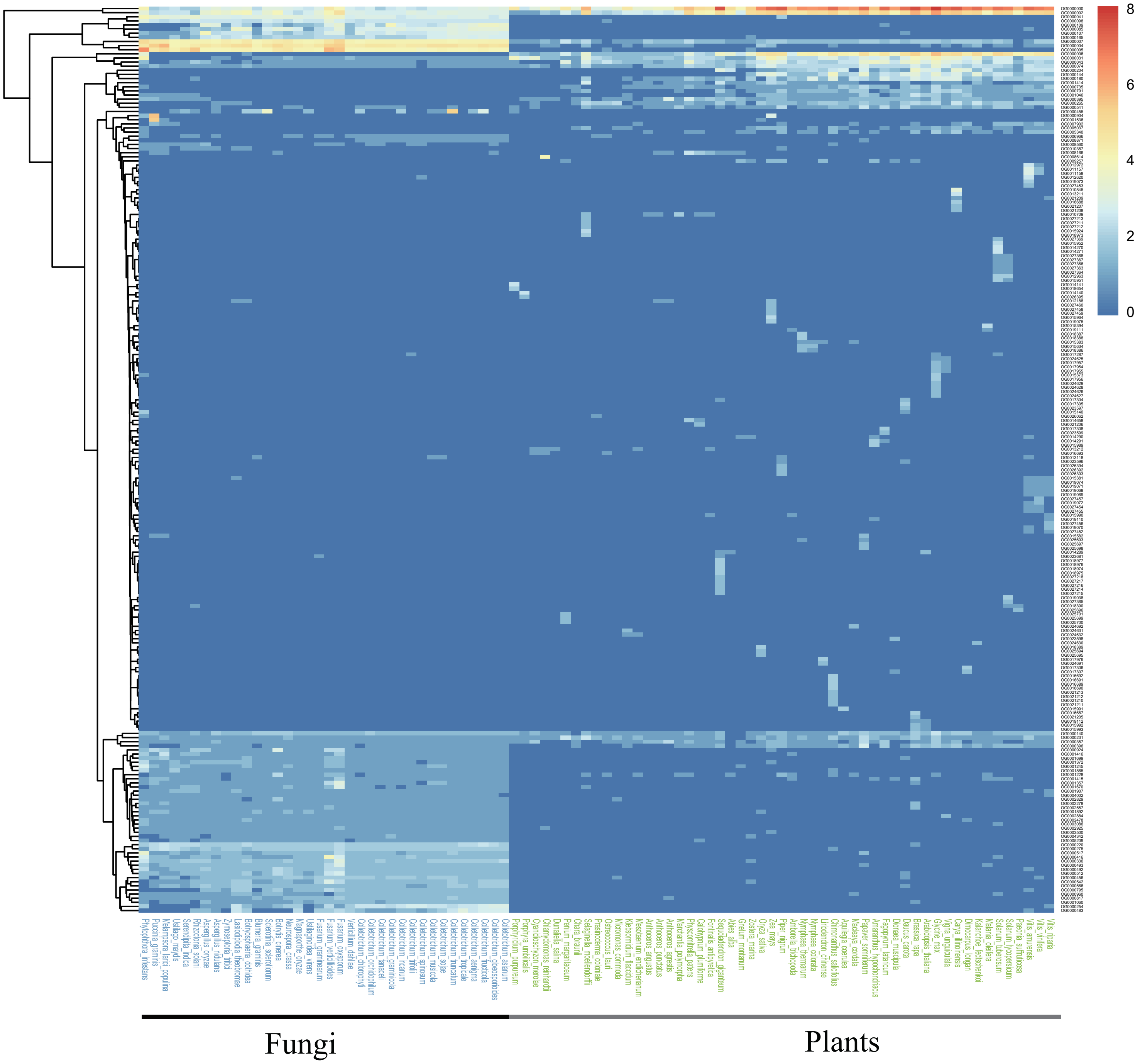

Figure S10 The heatmap of gene count in the 97 orthogroups including the homologous genes between fungi and plants. The orthogroups were identified using Orthofinder software. Blue cell represents low copy number, and red represents high copy number. Each row represents one orthogroup, and each column represents a plant species (blue text indicates fungi, and green text indicates plant species).

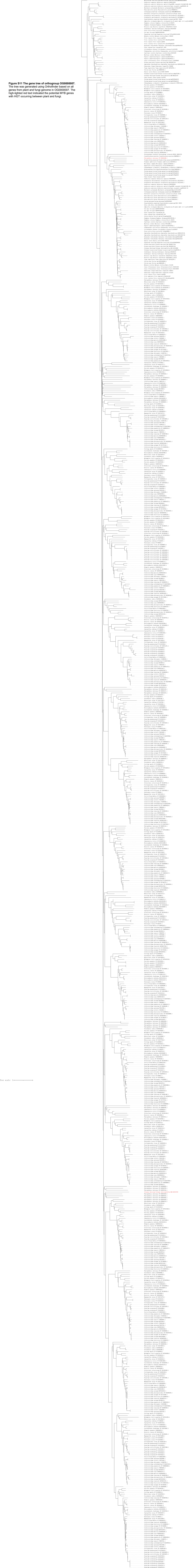

Figure S11 The gene tree of orthogroup OG0000007. The tree was generated using Orthofinder based on all genes from plant and fungi genome in OG0000007. The high-lighted red text indicated the potential MYB genes with HGT occurring between plant and fungi.

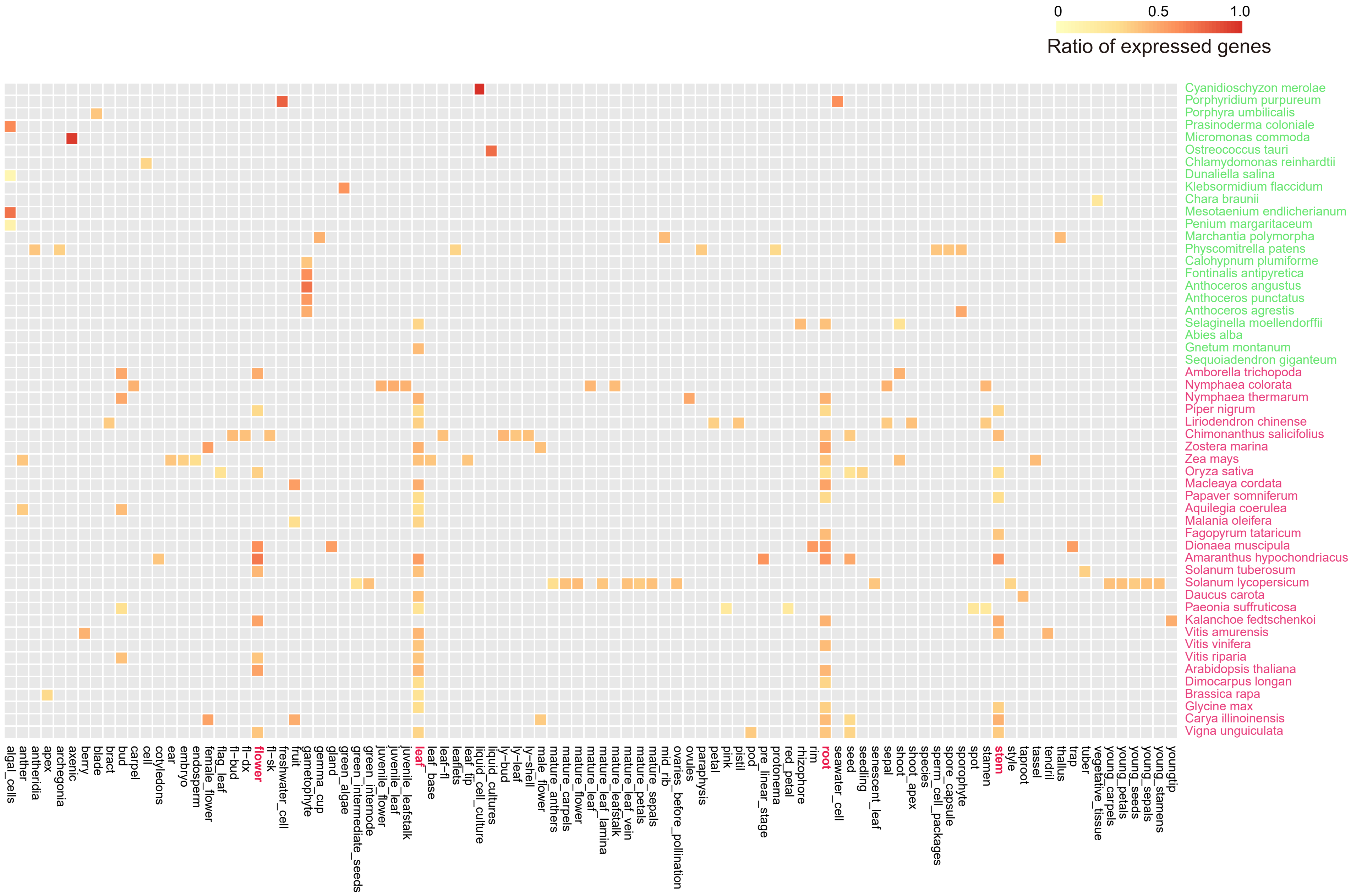

Figure S12 The heatmap of genes ratio (expressed gene number/total gene number) expressed (RPKM > 5) in different tissues from 53 selected plant species. Red font indi-cates the flowering plants, and green font indicates the non-flowering plants. Red cell indicates high ratio, yellow indicates low ratio, and grey indicates no expression. Each row represents a species, and each column represents one tissue (tissue with red font has much more RNA-seq data than the others).

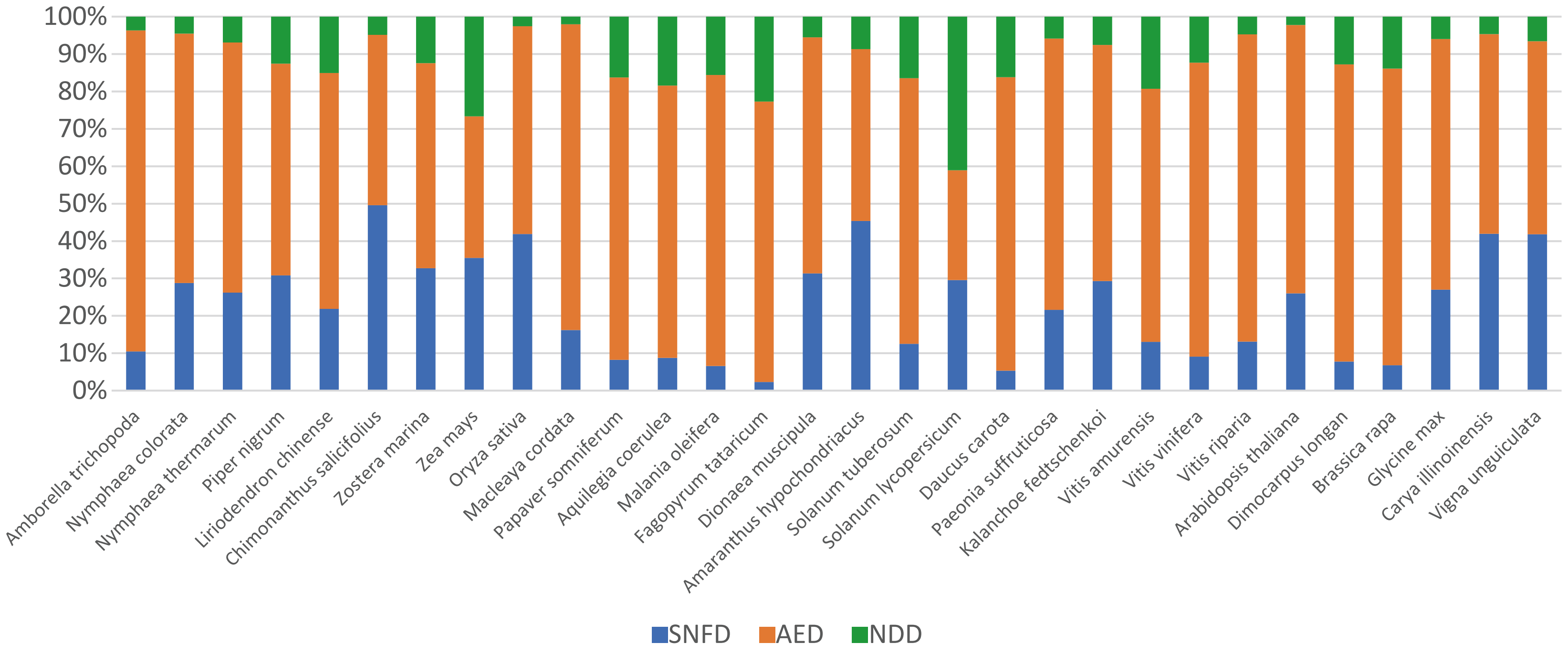

Figure S13 The ratio of gene duplicates belonging to sub-/neo-functionalized du-plicates (SNFD), asymmetrically Expressed Duplicates (AED), and no difference dupli-cates (NDD) categories in each of 30 flowering plants. Blue bar represents SNFD category, orange bar represents AED category, and green bar represents NDD category.

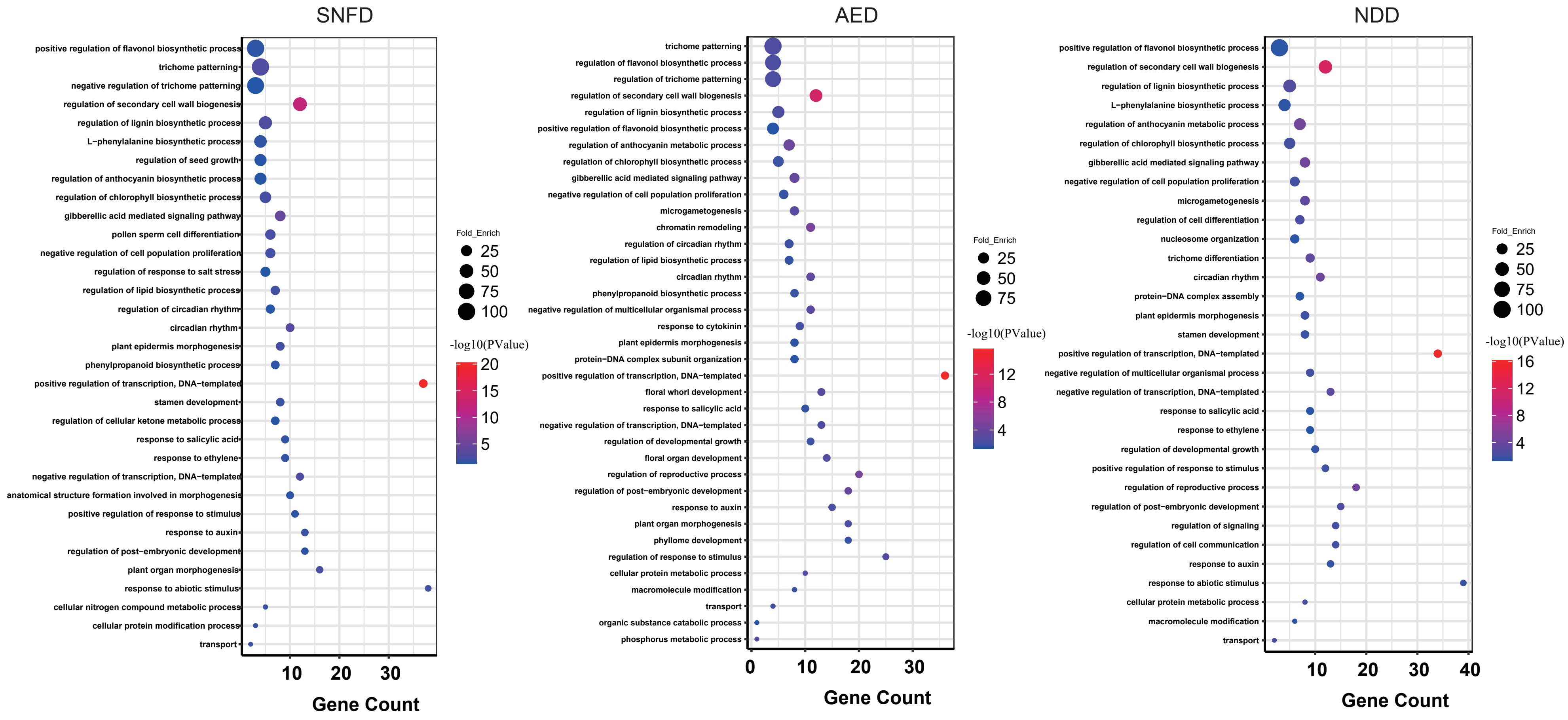

Figure S14 The GO enrichment of Arabidopsis homologous gene from SNFD, AED, and NDD categories. The bigger circle represented the higher fold enrichment and red bar represented lower *p*-value.

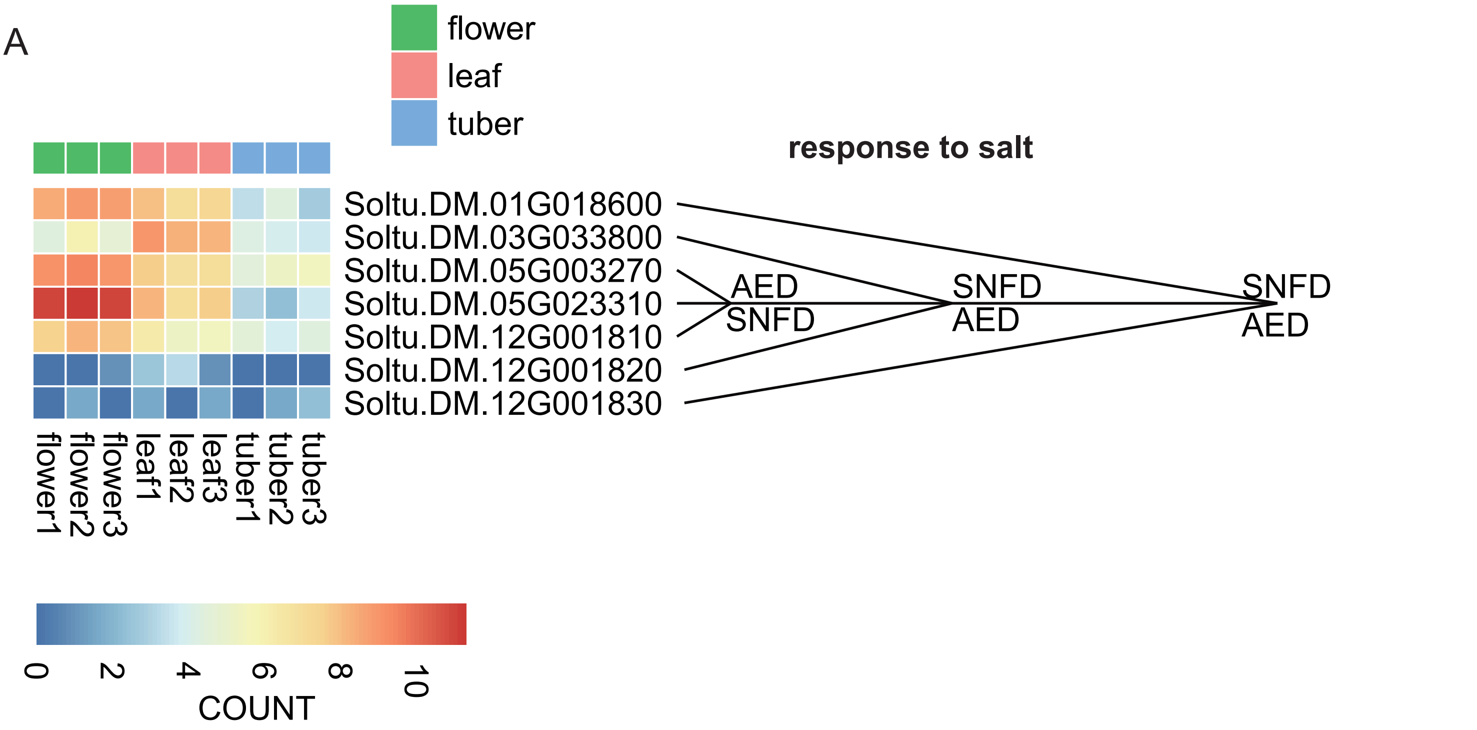

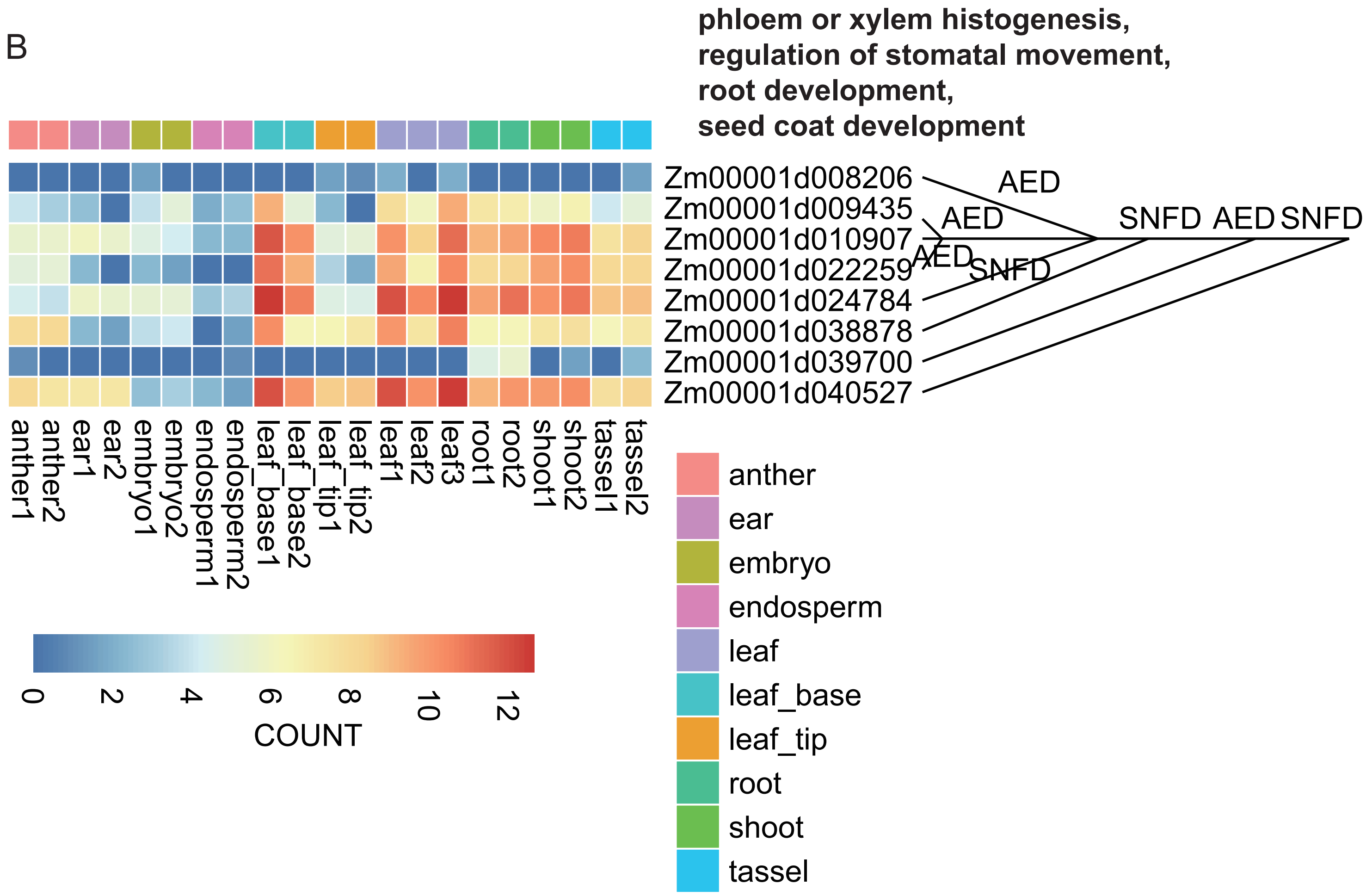

Figure S15 The expression pattern of gene duplicates in potato (A) and maize (B). Red color represents high expression and blue represents low expression. Different tissues were indicated using different color on the top. The right of heatmap indicates the gene types of SNFD, AED, or NDD beside the intersection of two lines.

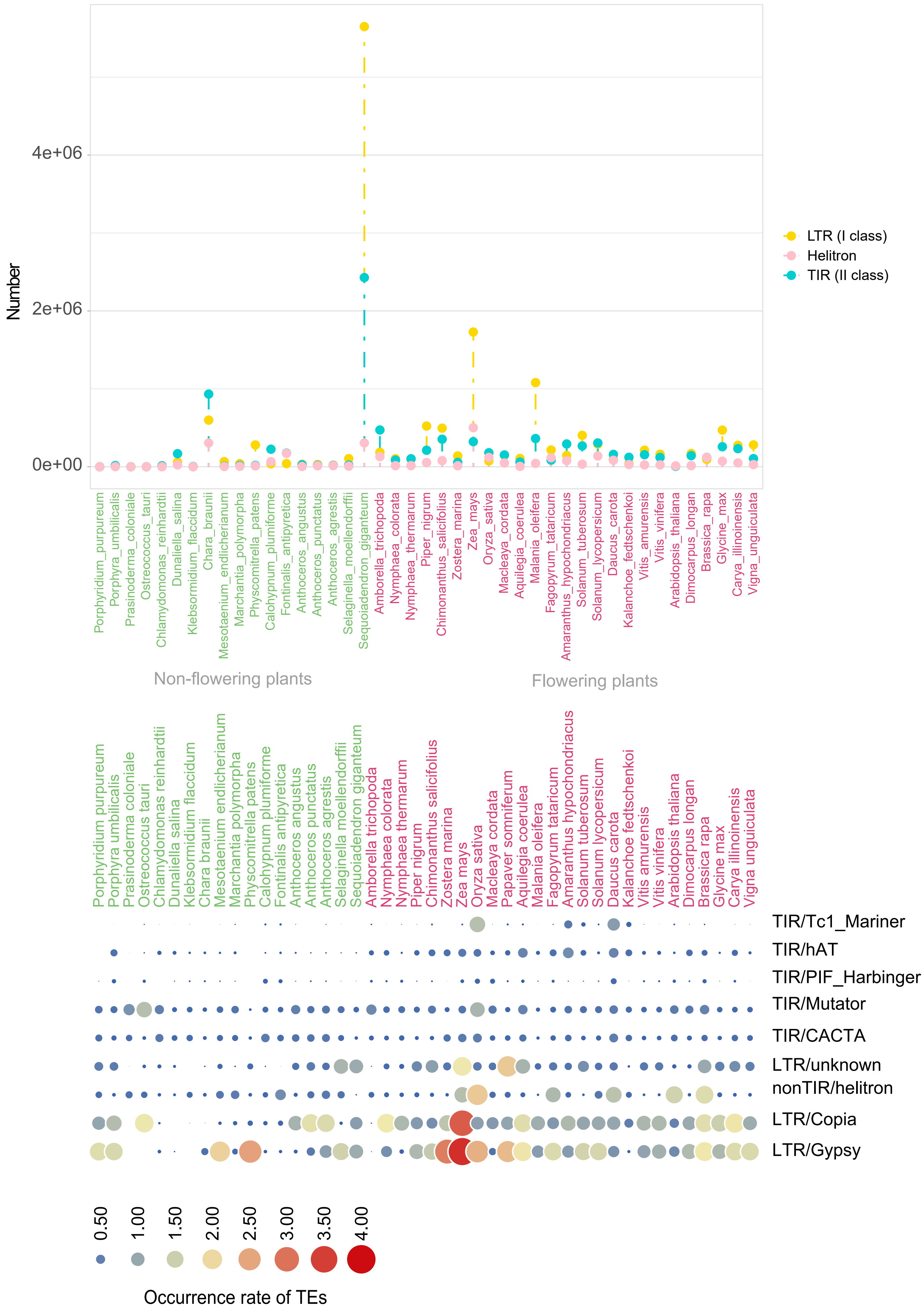

Figure S16 The number and occurring rate of different types of annotated TEs in 53 representative plant species from 22 categories. Green text represents non-flowering plants, and red text represents flowering plants. On the upper panel, y-axis represents the number of TEs, and y-axis represents the occurrence rate of TEs on the lower panel (bigger and red circles represent higher occurrence rate of TEs).

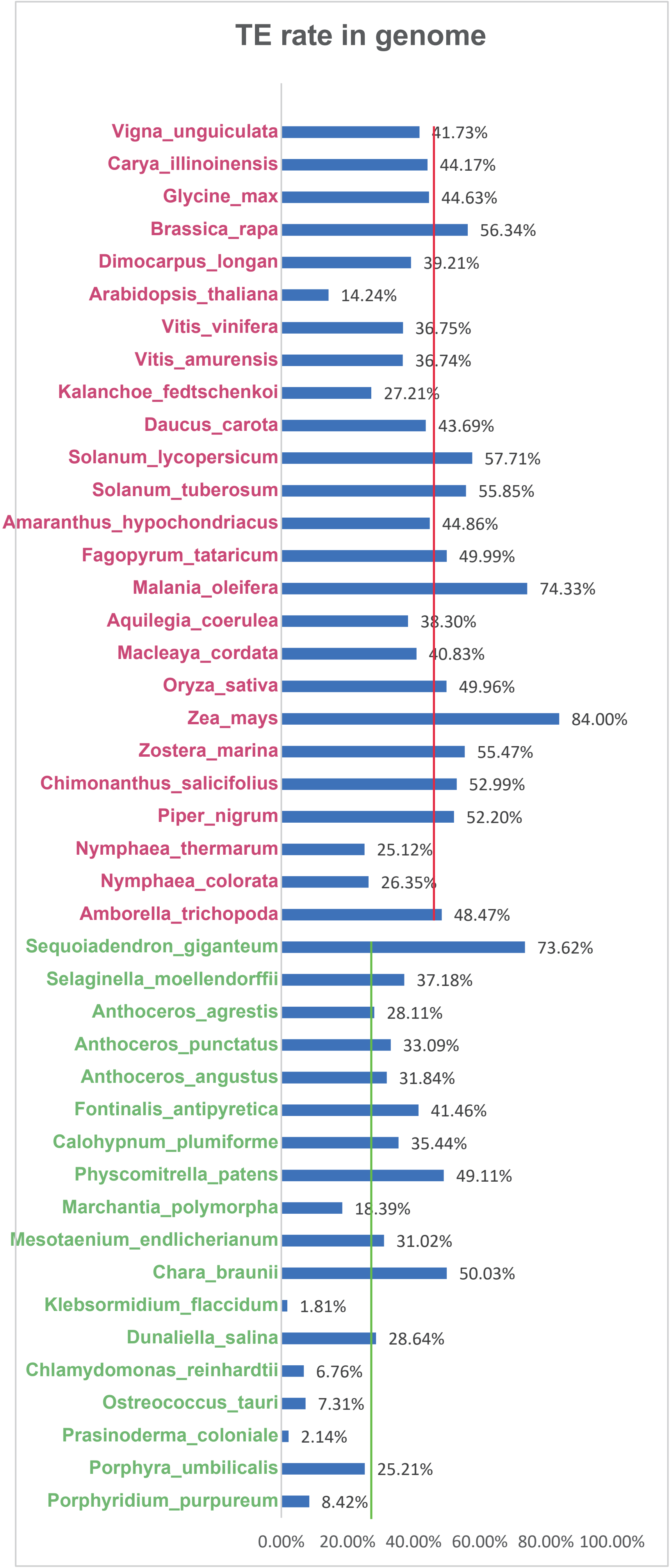

Figure S17 The rate of annotated TE in genomes of 53 representative plant species from 22 categories. Red and green line represented the average of TE rate in flowering and non-flowering plants, respectively.

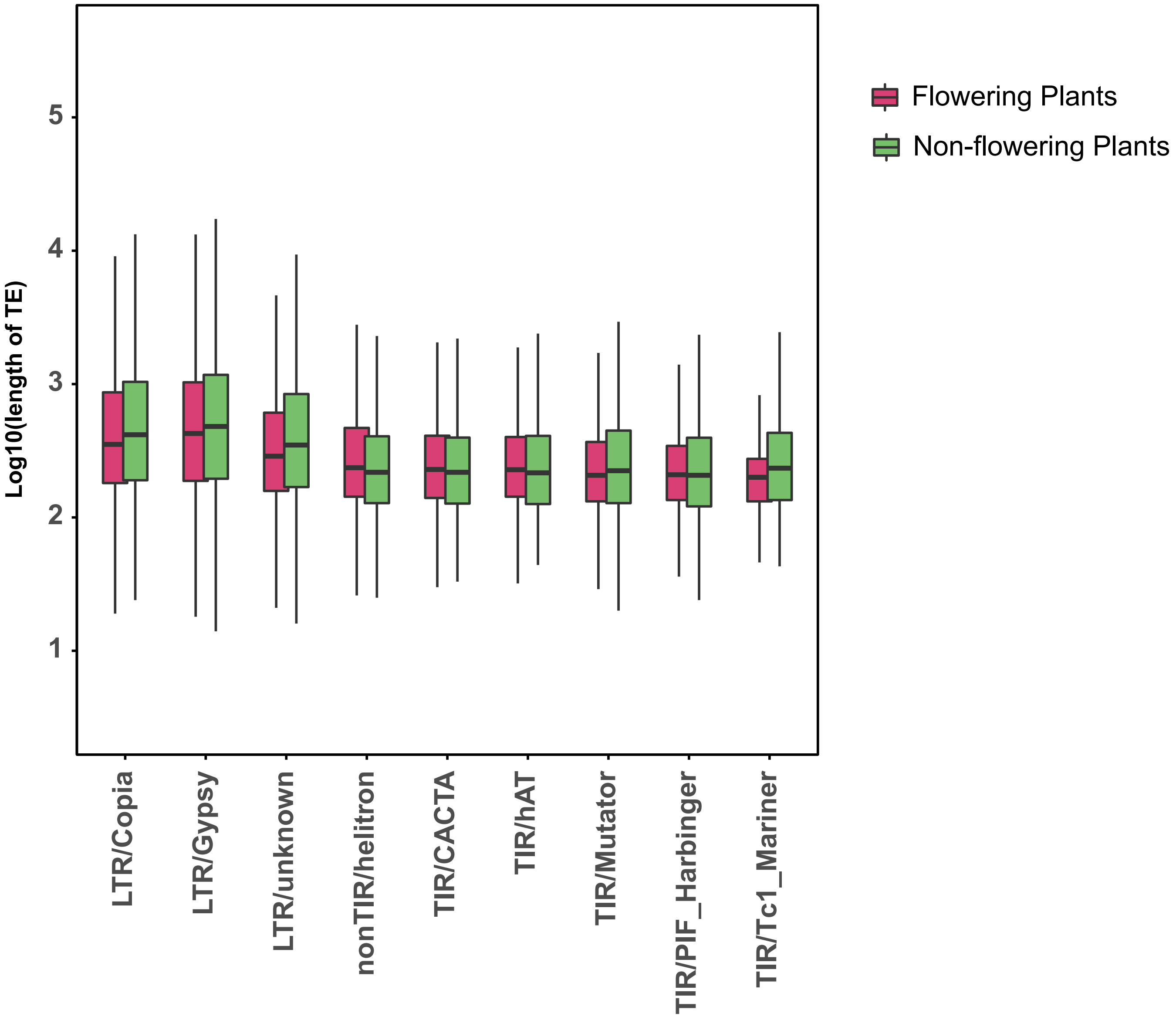

Figure S18 Boxplot displays the length of different types of annotated TE in flow-ering and non-flowering plant species. Red boxes represent the flowering plants, and green boxes represent the non-flowering plants. X-axis indicates the different types of TEs, and y-axis indicates the log10 transformed length of TEs.

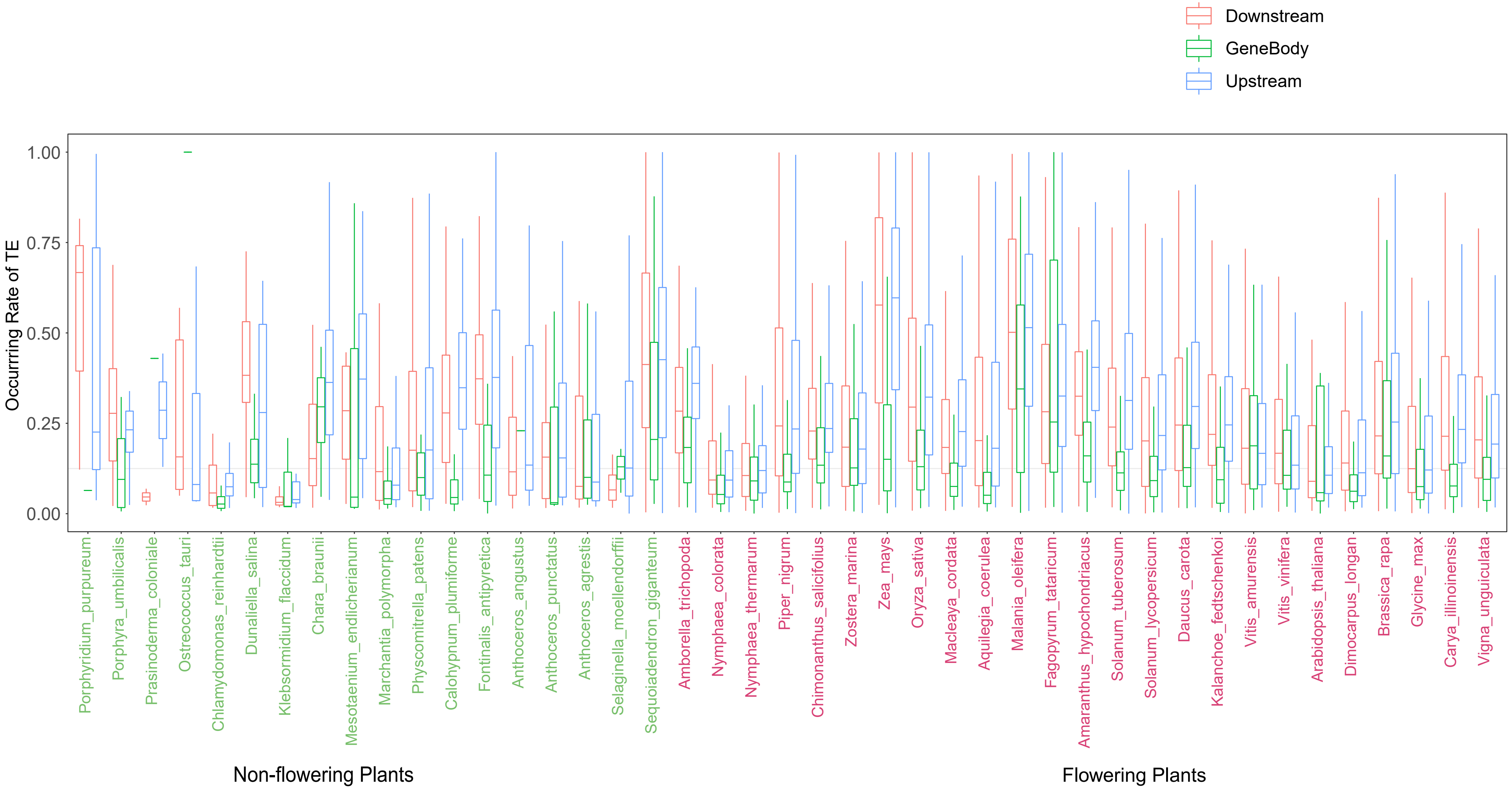

Figure S19 The occurring rate of TE on genic region and its up/downstream-5Kb re-gion of MYB genes in selected flowering and non-flowering plants. Red boxes represent the downstream regions, green boxes represent the gene body regions, and blue boxes represent the upstream regions. X-axis indicates different species (green text indicates the non-flowering plants, and red text represents the flowering plants), y-axis indicates the occurring rate of TEs.

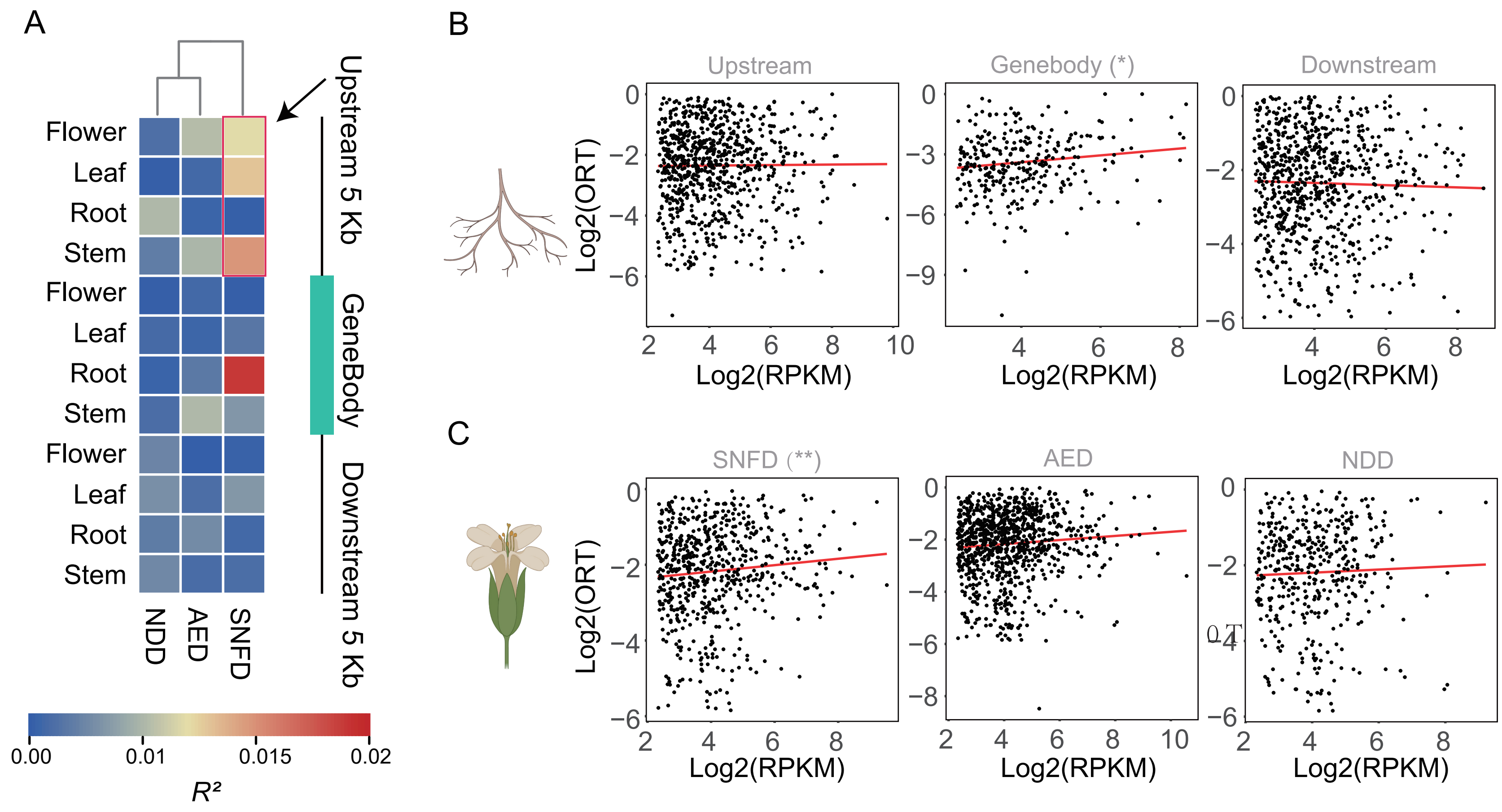

Figure S20 The correlation between occurrence rate of TEs in different genomic Regions and gene expression in different plant tissues.

(A) A heatmap showing a correlation between the occurrence rate of TEs (ORT) in dif-ferent genomic regions (upstream 5Kb, gene body, and downstream 5Kb) and gene ex-pression (RPKM > 5) in four different plant tissues. Red indicates a high correlation and blue indicates a low correlation.

(B) The correlation between MYB gene expression (RPKM) in root tissue and the occurrence rate of TEs in different genomic regions.

(C) The corre-lation between MYB gene expression (RPKM) in flower tissue and the occurrence rate of TEs in different categories (SNFD, AED, and NDD) of MYB genes. Values were log2 transformed. Double asterisk in the brackets indicates highly significant correlation.
